# The Endosymbiont Consortia of Two Cixiidae Planthoppers Reveal an Ancient Symbiosis With ‘*Candidatus* Mirabilia Symbiotica’

**DOI:** 10.1111/1758-2229.70204

**Published:** 2025-10-08

**Authors:** Jessica Dittmer, Mathieu Mahillon, Christophe Debonneville, Franco Faoro, Xavier Foissac, Olivier Schumpp, Bessem Chouaia

**Affiliations:** ^1^ Dipartimento di Scienze Agrarie e Ambientali Università degli Studi di Milano Milan Italy; ^2^ UMR 1345, Université d'Angers, Institut Agro, INRAE, IRHS, SFR Quasav Angers France; ^3^ Research Group Virology, Bacteriology and Phytoplasmology, Agroscope Nyon Switzerland; ^4^ Department of Plants and Crops Faculty of Bioscience Engineering, Ghent University Belgium; ^5^ UMR 1332 Biologie du Fruit et Pathologie, Université de Bordeaux, INRAE Bordeaux France

## Abstract

Insects of the suborder Auchenorrhyncha harbour multiple ancient endosymbionts that jointly produce essential nutrients lacking from the host's diet. Compared to cicadas, leafhoppers, and spittlebugs, our understanding of the multipartite symbioses among planthoppers, an extremely diverse insect group, is still very limited. Herein, we assembled the genomes of the primary endosymbionts of two planthopper species from the Cixiidae family, *Cixius wagneri* and *Pentastiridius leporinus*, both vectors of phytopathogenic *Arsenophonus* in Europe. Each species harboured a different tripartite endosymbiont consortium: while *P. leporinus* carried the well‐known combination ‘*Candidatus* Karelsulcia muelleri’, ‘*Ca*. Vidania fulgoroideae’, and ‘*Ca*. Purcelliella pentastirinorum’, *C. wagneri* harboured a yet unknown *Gammaproteobacterium* in addition to *Karelsulcia* and *Vidania*. This new endosymbiont ‘*Ca*. Mirabilia symbiotica’ is likely much older than *Purcelliella*, considering its extremely reduced genome. In both species, *Karelsulcia* and *Vidania* jointly produce the 10 essential amino acids, whereas *Purcelliella* and *Mirabilia* provide the non‐essential amino acid cysteine and slightly different gene sets encoding B vitamins. Our findings confirm the functional stability of multipartite planthopper endosymbiont consortia despite changing partners over evolutionary time. In addition, we describe a new *Rickettsia* strain from the Meloidae group colonising *P. leporinus* , highlighting the diversity of bacterial endosymbionts associated with planthoppers.

## Graphical abstract

Many insects harbour ancient beneficial endosymbionts that produce essential nutrients. Two planthopper species from the Cixiidae family harbour different tripartite endosymbiont consortia, including a previously unknown and likely ancient Gammaproteobacterium in addition to *Karelsulcia* and *Vidania*. The endosymbiont consortia have similar metabolic repertoires, maintaining functional stability despite changing partners.

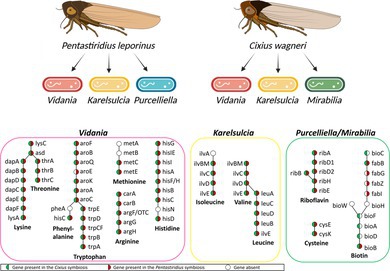

## 1 Introduction

Symbiotic associations with microorganisms have been essential for the evolution and diversification of insects since they enabled the exploitation of new ecological niches. Notably, some of the most intimate and long‐lasting symbioses can be found in insects thriving on nutrient‐poor diets such as plant sap or vertebrate blood (Baumann 2005; Buchner 1953). Their endosymbionts (mostly bacteria but also fungi) are crucial for host survival and reproduction since they provide essential amino acids and/or vitamins absent from the host's diet (Hansen and Moran 2011; Reis et al. 2020; Akman et al. 2002; Novakova et al. 2015; Dittmer et al. 2023; McCutcheon and von Dohlen 2011). The importance of these primary endosymbionts for the host is further exemplified by the evolution of specific symbiont‐housing organs and cells (bacteriomes/mycetomes and bacteriocytes/mycetocytes for bacteria and fungi, respectively) and transovarial transmission mechanisms (Buchner 1953; Michalik et al. 2021, 2023; Huang et al. 2020).

One of the most ancient obligate intracellular symbioses occurs in the Hemipteran suborder Auchenorrhyncha, which established a nutritional symbiosis with ‘*Candidatus* Karelsulcia muelleri’ (hereafter *Karelsulcia*, Bacteroidota) at least 260 million years ago, before the split of the Auchenorrhyncha into the Fulgoromorpha (planthoppers) and Cicadomorpha (cicadas, leafhoppers, treehoppers, and spittlebugs) (Moran et al. 2005; Müller 1962). All hosts of *Karelsulcia* feed on phloem or xylem sap, a food source deficient in essential amino acids, which are partly provided by the bacterium. However, such a strictly host‐associated lifestyle over hundreds of millions of years has profound implications for bacterial genome evolution. Indeed, obligate endosymbionts have small effective population sizes and experience strong transmission bottlenecks as well as relaxed selection on genes that are no longer required. This results in the accumulation of deleterious mutations and the eventual loss of unnecessary genes (McCutcheon and Moran 2012; Toft and Andersson 2010; McCutcheon et al. 2019). Over time, this process has produced the smallest bacterial genomes known to date, enriched in the nutritional biosynthetic pathways beneficial to the host (Bennett and Moran 2013). Eventually, even these essential metabolic pathways can be affected by ongoing genome erosion, leading to the acquisition of new, more metabolically versatile symbionts to complement or replace the decaying ones (Dittmer et al. 2023; Koga et al. 2013; Koga and Moran 2014; Monnin et al. 2020; Matsuura et al. 2018; Manzano‐Marin et al. 2017).

All these dynamics are at work in the Auchenorrhyncha: *Karelsulcia* has highly reduced genomes (142–287 Kbp, 137–264 protein‐coding genes) and, despite screening hundreds of insect species, has so far never been observed alone, but only in the company of one or more co‐primary endosymbionts. Indeed, the pioneering work of Buchner and Müller based on the light microscopy observation of over 400 species revealed that 55% and 30.5% of these species harboured two or three bacteriome‐associated endosymbionts, respectively, and a small number of species even harboured up to six symbionts (Buchner 1953; Müller 1962). Moreover, *Karelsulcia* was observed only in 64% of species but had been lost and replaced in the others (mainly in the Fulgoromorpha) by fungal symbionts and/or the so‐called ‘f‐symbiont’.

In the last 20 years, these observations have been largely confirmed using molecular and genomic techniques, shedding light on the taxonomy of some of the partner symbionts and their metabolic complementarities with *Karelsulcia*. Generally, *Karelsulcia* and a co‐primary symbiont jointly produce the 10 essential amino acids (EAAs) that the host requires, but with different nutritional roles depending on the insect host: hence, *Karelsulcia* can produce three, seven, or eight EAAs, and its co‐symbiont produces the remaining ones (Michalik et al. 2021; Bennett and Moran 2013; Wu et al. 2006; McCutcheon and Moran 2007, 2010; McCutcheon et al. 2009; Mao et al. 2017; Ankrah et al. 2018; Bennett and Mao 2018; Gossett et al. 2023; Deng et al. 2023). The identity of the co‐symbiont is also variable depending on the host species, the most widespread being the *Betaproteobacteria* ‘*Ca*. Zinderia insecticola’ (in spittlebugs), ‘*Ca*. Nasuia deltocephalinicola’ (in leafhoppers and treehoppers), and ‘*Ca*. Vidania fulgoroideae’ (hereafter *Vidania*, in planthoppers) as well as the *Alphaproteobacterium* ‘*Ca*. Hodgkinia cicadicola’ in cicadas. These ancient co‐symbionts have highly eroded genomes themselves due to long co‐evolutionary histories with their insect hosts (McCutcheon et al. 2009; Deng et al. 2023; Vasquez and Bennett 2022; Urban and Cryan 2012; Gonella et al. 2011; Van Leuven et al. 2014) and have been replaced or complemented by other bacteria or fungi in various lineages (Koga et al. 2013; Koga and Moran 2014; Matsuura et al. 2018; Sacchi et al. 2008; Kobialka, Michalik, Szwedo, and Szklarzewicz 2018; Kobialka, Michalik, and Szklarzewicz 2018; Kobialka, Michalik, Walczak, and Szklarzewicz 2018; Kobialka et al. 2019).

The endosymbiont consortia of planthoppers are even more complex in terms of the number of partners, since *Karelsulcia* and *Vidania* are often accompanied by a third bacteriome‐associated endosymbiont that provides B vitamins. These additional symbionts have so far been identified as ‘*Ca*. Purcelliella pentastirinorum’ (hereafter *Purcelliella*), *Sodalis*, and *Arsenophonus* (Michalik et al. 2021, 2023; Bennett and Mao 2018; Gossett et al. 2023; Deng et al. 2023; Gonella et al. 2011; Bressan, Arneodo, et al. 2009). Whereas *Sodalis* and *Arsenophonus* have been observed in several planthopper families (Michalik et al. 2023), *Purcelliella* has only been observed in species belonging to the Cixiidae family (Michalik et al. 2023; Bennett and Mao 2018; Gossett et al. 2023; Bressan, Arneodo, et al. 2009) and likely corresponds to Müller's ‘c + d symbiont’, due to its localisation in at least two different bacteriomes (Buchner 1953; Müller 1962; Bressan and Mulligan 2013). Considering their larger genomes (480 Kbp for *Purcelliella*, several Mbp for *Sodalis* and *Arsenophonus*), smaller host range, and apparent supporting nutritional role, these symbionts were likely acquired much more recently. Moreover, *Purcelliella* genomes have retained different sets of B vitamins depending on the ecological context, suggesting variation in selection pressure and adaptation to different ecological niches (Gossett et al. 2023). In addition to the primary endosymbionts, planthoppers can also harbour other common insect symbionts such as *Rickettsia*, *Wolbachia*, or *Cardinium* (Michalik et al. 2023). Their functional roles have not yet been elucidated and may be facultative for the insect host, but they have sometimes been observed in unusual localisations (e.g., within the host nuclei or in the cytoplasm of another bacterium (Michalik et al. 2023; Arneodo et al. 2008; Michalik et al. 2024)) and can share the bacteriocytes with *Karelsulcia* or *Vidania* (Michalik et al. 2023). This raises the question of whether they could eventually replace one of the more ancient endosymbionts, as already observed for *Arsenophonus* and *Sodalis* (Michalik et al. 2023).

Despite the growing interest in recent years, our understanding of the multipartite symbioses among planthoppers, an extremely diverse insect group, is still limited, and genomic data are available for only a handful of species. Herein, we investigated the endosymbiont consortia of two planthopper species from the Cixiidae family, *Cixius wagneri* and *Pentastiridius leporinus*. Both species are vectors of phytopathogenic *Arsenophonus* bacteria in several European countries (France, Italy, Switzerland, Germany) (Bressan 2014; Mahillon et al. 2022; Behrmann et al. 2023; Terlizzi et al. 2007; Danet et al. 2003; Semetey et al. 2007; Salar et al. 2010), and metagenomic data had been initially obtained for a comparative genomics analysis of the phytopathogens (Mahillon et al. 2025). Assembling the genomes of additional endosymbionts from the same data revealed that the two species harboured different tripartite primary endosymbiont consortia: While *P. leporinus* carried the already well‐known combination *Karelsulcia*, *Vidania*, and *Purcelliella*, *C. wagneri* harboured a yet unknown *Gammaproteobacterium* in addition to *Karelsulcia* and *Vidania*. This new endosymbiont, for which we propose the name ‘*Ca*. Mirabilia symbiotica’, is likely much older than *Purcelliella* considering its extremely reduced genome and could correspond to Müller's ‘b symbiont’. In addition, we describe a new *Rickettsia* strain from the Meloidae group colonising *P. leporinus* and investigate the nutritional complementarities within the different endosymbiont consortia.

## 2 Materials and Methods

### 2.1 Planthopper Samples

Adult specimens of *C. wagneri* were collected with a sweep net in a strawberry field in the Dordogne region (France) in June 2019. Adult *P. leporinus* collected in the Burgundy region (France) in the early 2000s were kindly provided by Frédéric Gatineau (Cirad). Additional *P. leporinus* specimens were collected in a sugar beet field in Gilly (Switzerland) in 2020 using an SH 86 suction device (Stihl). The sampling sites correspond to outbreak areas of Marginal Chlorosis disease of strawberry caused by ‘*Ca*. Phlomobacter fragariae’ (Danet et al. 2003) or the sugar beet disease ‘syndrome basses richesses’ caused by ‘*Ca*. Arsenophonus phytopathogenicus’ (Mahillon et al. 2022; Gatineau et al. 2002). DNA was extracted from individual insects using established CTAB protocols (Mahillon et al. 2022, 2025).

### 2.2 Metagenome Sequencing

Long‐read metagenome sequencing using an Oxford Nanopore–Illumina hybrid approach was performed on single individuals of *C. wagneri* from France (CW) and *P. leporinus* collected in Switzerland (PLCH). Since the *P. leporinus* specimens from France had been stored for more than 15 years prior to DNA extraction, their DNA was highly fragmented, precluding long‐read sequencing. Therefore, the metagenome of a *P. leporinus* specimen from France (PLFR) was sequenced using Illumina technology only. Long‐read sequencing libraries were prepared using the ligation sequencing kits SQK‐LSK 109 and SQK‐LSK 110 (Oxford Nanopore Technologies, UK) for CW and PLCH, respectively. Each library was sequenced on an entire R9.4 flow cell on the MinION sequencer, producing 6 and 16 Gbases of data, respectively. Basecalling was done using Guppy v5.0.11 in high‐accuracy mode, and low‐quality (< Q7) as well as short (< 500 bp) reads were discarded. In addition, 2 × 150 bp paired‐end reads were obtained from an Illumina Novaseq platform (Macrogen Europe), producing 476, 486, and 50 million reads for CW, PLFR, and PLCH, respectively. The Illumina reads were quality‐trimmed using Trimmomatic v0.38 (Bolger et al. 2014), retaining only reads ≥ Q30.

### 2.3 Primary Endosymbiont Genome Assembly

To discard host reads, the Illumina reads were assembled using Megahit v1.2.9 (Li et al. 2015) and all contigs belonging to Hemiptera were identified using BlobTools v1.1.1 (https://github.com/DRL/blobtools). These contigs were then used as reference to remove host reads from the long‐read datasets via mapping with Minimap2 v2.15 (Li 2018). The remaining non‐host reads ≥ 500 bp were assembled using different methods: (1) Assembly of Nanopore reads using Flye v2.8.1 (Kolmogorov et al. 2020) with the options ‐‐nano‐raw and ‐‐meta, and (2) a Nanopore‐Illumina hybrid assembly using Unicycler v0.4.9 (Wick et al. 2017). Contigs belonging to the primary endosymbionts were identified using Blast (Altschul et al. 1990). The most contiguous assemblies were obtained with Flye for CW and with Unicycler for PLCH. For PLCH, Unicycler produced three circular contigs corresponding to the complete genomes of ‘*Ca*. Karelsulcia muelleri’, ‘*Ca*. Vidania fulgoroideae’, and ‘*Ca*. Purcelliella pentastirinorum’. For CW, Flye produced two circular contigs corresponding to the genomes of *Vidania* and an unknown *Gammaproteobacterium* as well as nine contigs belonging to *Karelsulcia*. All assemblies from CW were polished with Nanopore reads using Medaka v1.5.0 (https://github.com/nanoporetech/medaka) and subsequently with Illumina reads using several iterations of Polca from the MaSuRCa toolkit v4.0.7 (Zimin and Salzberg 2020), until no more errors were found. Assembly quality was verified by mapping the reads against the circular genome assemblies (using Minimap2 v2.15 (Li 2018) for long reads and BWA v0.7.17 (Li and Durbin 2010) for short reads) and checking for consistent coverage in the IGV genome browser (https://igv.org/app/). The completeness and contamination level of each assembly were assessed using CheckM2 v0.1.3 (Chklovski et al. 2023) and compared to CheckM2 values of the corresponding endosymbionts from *Oliarus filicicola* OLIH as reference (Bennett and Mao 2018).

Although the total size of the *Karelsulcia* assembly from CW was comparable to other *Karelsulcia* genomes from Fulgoromorpha (154 Kbp), CheckM2 v0.1.3 (Chklovski et al. 2023) predicted a low level of completeness (47.95%) and a high level of contamination (4.15%). An alternative long‐read assembly using Canu v2.1.1 (Koren et al. 2017) produced similar results. Therefore, we extracted all reads belonging to *Karelsulcia* from both the Nanopore and Illumina datasets from CW via mapping against the closed genome of the *Karelsulcia* strain OLIH (Genbank accession GCF_003391295.1) and performed a hybrid assembly using SPAdes v3.15.1 (Prjibelski et al. 2020). This produced 82 contigs which could be scaffolded into 38 contigs using Redundans v0.14 (Pryszcz and Gabaldon 2016), SSPACE v2.1.1 (Boetzer et al. 2011), and Gapfiller v2.1.2 (Boetzer and Pirovano 2012). Despite remaining incomplete (the final assembly size was 129 Kbp), this assembly had high completeness (81.99%) and low contamination (0.20%), comparable to the *Karelsulcia* OLIH genome (83.91% completeness, 0.18% contamination).

The endosymbiont genomes from PLFR were assembled from Illumina short reads using Megahit v1.2.9 (Li et al. 2015), resulting in 7 contigs for *Karelsulcia*, 6 contigs for *Vidania*, and 23 contigs for *Purcelliella*. These contigs were manually reordered based on the complete endosymbiont genomes from PLCH, and all three genomes could be closed by scaffolding using Redundans v0.14 (Pryszcz and Gabaldon 2016).

### 2.4 Genome Assembly of *Rickettsia* Strains From *P. leporinus*

The metagenomes from both species contained contigs belonging to the common insect endosymbiont *Wolbachia*. In addition, both PLCH and PLFR assemblies also contained numerous contigs belonging to *Rickettsia*. The coverage of the *Wolbachia* contigs was so low that we did not attempt any draft assemblies, but we obtained a high‐quality draft genome of the *Rickettsia* strain from PLCH. To do so, all Nanopore and Illumina reads from PLCH mapping onto *Rickettsia* contigs from the initial Unicycler assembly were extracted from the dataset. Since the long‐read coverage alone was insufficient for a *de novo* assembly using Flye, we again performed hybrid assemblies using SPAdes v3.15.1 (Prjibelski et al. 2020) and Unicycler v0.4.9 (Wick et al. 2017). The most contiguous assembly with the highest completeness based on CheckM2 was obtained using SPAdes. *Rickettsia* reads were then extracted from the PLFR Illumina data via mapping against the *Rickettsia* assembly from PLCH and assembled using SPAdes v3.15.1 (Prjibelski et al. 2020). The resulting contigs were scaffolded using two rounds of Redundans v0.14 (Pryszcz and Gabaldon 2016).

### 2.5 Functional Genome Analyses

All genome assemblies were annotated using the NCBI Prokaryotic Genome Annotation Pipeline (PGAP) version 2023‐10‐03.build7061 (Tatusova et al. 2016). Synteny plots between the endosymbionts from CW and PL as well as from *O. filicicola* OLIH (Bennett and Mao 2018) as reference were produced using the pyGenomeViz v0.4.4 web application (https://pygenomeviz.streamlit.app). For visualisation purposes, the 38 contigs of the *Karelsulcia* strain from CW were reordered against the complete *Karelsulcia* genome from PLCH using progressive Mauve v20150226 (Darling et al. 2004). Average nucleotide identity (ANI) was calculated using the EZBioCloud ANI calculator (https://www.ezbiocloud.net/tools/ani). Clusters of Orthologous Genes (COG) categories were determined using eggNOG‐mapper v2.1.12 (Cantalapiedra et al. 2021) and KEGG pathway annotations were obtained from BlastKOALA v3.0 (Kanehisa et al. 2016). Symbiont genes involved in the biosynthesis of essential amino acids and B vitamins were identified based on the eggNOG‐mapper and KEGG pathway annotations as well as manual BlastX searches against the NCBI non‐redundant protein database. A heatmap showing KEGG pathway completeness of selected *Rickettsia* genomes was created using KEGGDecoder v1.3 (Graham et al. 2018).

### 2.6 Phylogenetic Analyses

Phylogenomic analyses were performed for each endosymbiont (i.e., *Karelsulcia*, *Vidania*, *Purcelliella*/*Gammaproteobacteria* endosymbiont of CW and *Rickettsia*). Genomes downloaded from NCBI for these analyses in May 2024 (Table S1) were reannotated with PGAP version 2023‐10‐03.build7061 (Tatusova et al. 2016) to avoid any biases due to different annotations. Orthofinder v2.5.4 (Emms and Kelly 2019) was then used to identify single‐copy orthologous genes shared (i) between 14 *Karelsulcia* genomes from Fulgoromorpha and seven additional *Karelsulcia* strains from Cicadomorpha as an outgroup, (ii) between 14 *Vidania* genomes from Fulgoromorpha and five *Nasuia* strains from Cicadomorpha as an outgroup, (iii) between all available *Purcelliella* genomes (*N* = 7), the *Gammaproteobacteria* endosymbiont of CW and 23 other nutritional endosymbionts from the *Gammaproteobacteria*, and (iv) 34 *Rickettsia* genomes spanning the genetic diversity of this genus based on (Davison et al. 2022) with five genomes from ‘*Ca*. Tisiphia’ spp., ‘*Ca*. Megaera polyxenophila’ strain SAG 25.80 and *Orientia tsutsugamushi* strain UT76 as an outgroup. The amino acid sequences of each conserved gene were aligned using Muscle v5.2 (Edgar 2004) and the alignments were concatenated into a partitioned supermatrix using the script geneStitcher.py (https://github.com/ballesterus/Utensils/blob/master/geneStitcher.py). IQ‐TREE v1.6.12 (Minh et al. 2020) was used to predict the optimal amino acid substitution model for each gene partition (Kalyaanamoorthy et al. 2017; Chernomor et al. 2016) and to produce a Maximum Likelihood phylogenetic tree with 1000 bootstrap iterations. The tree was visualised in FigTree v1.4.4 (https://github.com/rambaut/figtree).

An additional phylogenetic analysis was performed on the near‐complete 16S rRNA gene sequences of 41 *Gammaproteobacteria* endosymbionts, including *Purcelliella* symbionts from other planthoppers and an endosymbiont of *C. nervosus* (Accession: OQ099687.1) for which no genome sequence is available. As for the phylogenomic analyses, the sequences were aligned using Muscle, and IQ‐TREE was used to predict the optimal substitution model and to produce a Maximum Likelihood phylogenetic tree with 1000 bootstrap iterations.

## 3 Results

### 3.1 *P. leporinus* and *C. wagneri* Harbour Different Primary Endosymbiont Consortia

**FIGURE 1:**
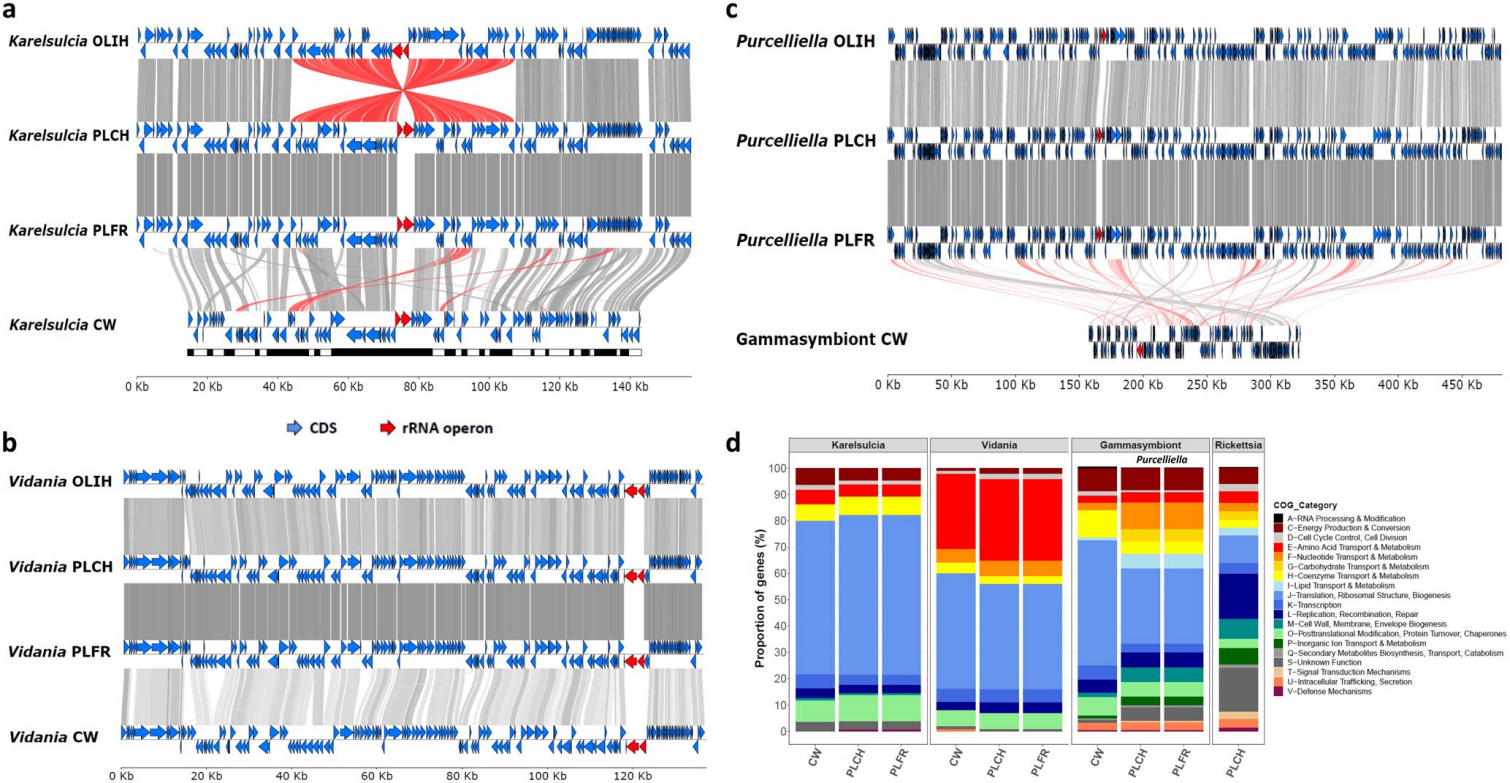
Comparison of primary endosymbiont genomes from different Cixiidae species. Synteny plots comparing the genomes of *Karelsulcia* (a), *Vidania* (b), and the *Gammaproteobacteria* endosymbionts (c) from *C. wagneri* and *P. leporinus* , including the corresponding endosymbionts from *O. filicicola* OLIH as reference. Protein‐coding genes are indicated in blue, ribosomal rRNA operons in red. Grey shading indicates the degree of pairwise sequence similarity between genomes; red shading indicates inversions. Contigs of the fragmented *Karelsulcia* CW genome are indicated as black and white boxes below the genes. (d) COG functional categories for the primary endosymbiont genomes.

Both *P. leporinus* specimens PLFR and PLCH sampled about 15 years apart in France and Switzerland, respectively, harboured the same endosymbiont consortium consisting of the three primary endosymbionts *Karelsulcia*, *Vidania*, and *Purcelliella*. All six genomes could be closed and the genome pairs from the two sequenced individuals were highly similar (Table 1). Specifically, the two *Karelsulcia* genomes were both 157,371 bp in length and differed by only two single‐nucleotide polymorphisms (SNPs) across the entire genome. The two *Vidania* genomes also had the same size (135,961 bp) and differed by 68 SNPs. The *Purcelliella* genome from PLCH was slightly longer than the one from PLFR (481,089 and 481,066 bp, respectively) with 122 SNPs between the two genomes. However, these differences were negligible as the gene content was completely identical in all endosymbiont genome pairs (Figure 1). Compared to endosymbiont genomes from *O. filicicola* OLIH, the first Cixiidae species that had its endosymbionts' genomes sequenced (Bennett and Mao 2018), the endosymbionts of *P. leporinus* are almost identical in size, gene content, and synteny, except for a large inversion between the *Karelsulcia* genomes from *P. leporinus* and *Karelsulcia* OLIH (Figure 1a). Moreover, comparing the orthogroups present in each endosymbiont species revealed that 97%, 98%, and 100% of orthogroups present in *Purcelliella*, *Vidania*, and *Karelsulcia* from *P. leporinus* , respectively, were shared with their endosymbiont counterparts from *O. filicicola* and/or *O. polyphemus* (Figure S1).

**TABLE 1:** Summary of the endosymbiont genomes obtained in this study.

Symbiont	Host species	Origin	Contigs	Length (bp)	GC%	Genes	CDS	Pseudogenes	rRNAs	tRNAs	ncRNAs	Completeness^a^
*Karelsulcia muelleri* CW	*Cixius wagneri*	France	38	128,919	26.7	140	111	10	3	14	2	81.99
*Karelsulcia muelleri* PLFR	*Pentastiridius leporinus*	France	1	157,371	25.4	183	148	1	3	29	2	85.81
*Karelsulcia muelleri* PLCH	*Pentastiridius leporinus*	Switzerland	1	157,371	25.4	183	148	1	3	29	2	85.81
*Vidania fulgoroideae* CW	*Cixius wagneri*	France	1	137,240	21.5	179	152	0	3	24	0	83.93
*Vidania fulgoroideae* PLFR	*Pentastiridius leporinus*	France	1	135,961	19.0	185	158	0	3	24	0	74.38
*Vidania fulgoroideae* PLCH	*Pentastiridius leporinus*	Switzerland	1	135,961	19.0	185	158	0	3	24	0	74.38
Gammaproteobacteria symbiont CW	*Cixius wagneri*	France	1	165,642	18.4	261	181	52	3	25	0	68.89
*Purcelliella pentastirinorum* PLFR	*Pentastiridius leporinus*	France	1	481,066	21.7	470	427	7	3	31	2	99.25
*Purcelliella pentastirinorum* PLCH	*Pentastiridius leporinus*	Switzerland	1	481,089	21.7	470	427	7	3	31	2	99.25
*Rickettsia* PLFR	*Pentastiridius leporinus*	France	130	1,304,041	33.2	1412	1232	147	2	27	4	86.76
*Rickettsia* PLCH	*Pentastiridius leporinus*	Switzerland	107	1,338,603	33.3	1414	1272	107	3	28	4	86.76

^a^ Based on CheckM2 v0.1.3.

In contrast, the primary endosymbiont consortium of *C. wagneri* was different: While this species also harboured *Karelsulcia* and *Vidania* (Table 1 and Figure 1a,b), *Purcelliella* was not detected. Instead, we assembled the circular genome of another, yet uncharacterised *Gammaproteobacteria* symbiont. Its coverage of 80× was the second highest in the Nanopore data, after *Vidania* at 200×. Notably, this genome was much smaller compared to *Purcelliella* (165,642 bp), had an even lower GC content (Table 1), and completely lacked synteny with *Purcelliella* (Figure 1c). Its functional repertoire based on COG categories was also different from *Purcelliella*, with a larger fraction of genes dedicated to ‘Translation and ribosomal structure’ (47.54% vs. 28.64% in *Purcelliella*), similar to the more reduced endosymbionts *Vidania* and *Karelsulcia* (Figure 1d). The category ‘Coenzyme transport and metabolism’ was also more highly represented in the *Gammaproteobacteria* symbiont compared to *Purcelliella* (10.38% vs. 4.54%), whereas numerous other categories (i.e., ‘Nucleotide transport and metabolism’, ‘Carbohydrate transport and metabolism’, ‘Lipid transport and metabolism’, ‘Cell wall, membrane and envelope biogenesis’, and ‘Inorganic ion transport and metabolism’) were less represented in the *Gammaproteobacteria* symbiont compared to *Purcelliella* (Figure 1d).

In contrast to the other endosymbiont genomes, PGAP predicted an unusually high number of short protein‐coding genes (CDS) and pseudogenes for the new *Gammaproteobacteria* endosymbiont from *C. wagneri* (Table 1). Manual inspections of all predicted CDS of this genome using BlastX conserved domain searches against the NCBI non‐redundant protein database revealed that 87 genes were split in two halves. Manually merging the two parts generally reconstituted the complete functional domain in frame, but with a stop codon within the coding sequence. A new annotation with the coordinates of the merged genes is provided in Table S1. Considering the high and even coverage of this genome and the fact that no other assembly presented this problem, we consider it unlikely that this is due to sequencing or assembly errors. This leaves the question of whether the split genes are indeed pseudogenized or whether this symbiont might have a different codon usage where some stop codons have been repurposed, maybe as a result of the strong AT bias. To investigate this hypothesis, we analysed the stop codons for three groups of genes: (1) genes without an internal stop codon, (2) the first half of split genes until the internal stop codon, and (3) the second half of split genes after the internal stop codon. This revealed that stop codon usage was indeed somewhat different between the three groups. Whereas groups 1 and 3 used mainly TAA (group 1: 80% TAA, 13% TGA, 7% TAG/group 3: 71% TAA, 24% TGA, 5% TAG), the internal stop codons used TGA at a higher frequency compared to the other groups (55% TAA, 36% TGA, 9% TAG). However, further research will be needed to truly elucidate whether the split genes are still functional or not.

Considering its typical hallmarks of a long endosymbiotic association, such as extremely small genome size, low GC content, and reduced metabolic capabilities, we hypothesise that the *Gammaproteobacteria* symbiont is the third primary endosymbiont of *C. wagneri* , analogous to *Purcelliella* in *P. leporinus* . Interestingly, the species *C. nervosus* had been included in a recent large‐scale planthopper endosymbiont screening based on 16S rRNA gene sequences and was reported to harbour *Purcelliella* as well as *Karelsulcia* and *Vidania* (Michalik et al. 2023). To verify whether our newly discovered endosymbiont might be specific to *C. wagneri* , we compared the 16S rRNA gene sequence identity between the *C. nervosus* endosymbiont (Accession: OQ099687.1), the *C. wagneri* endosymbiont, and *Purcelliella* PLCH. This revealed that the *C. nervosus* endosymbiont shared 96% 16S rRNA gene sequence identity with the *C. wagneri* endosymbiont, but only 84% sequence identity with *Purcelliella*, indicating that the initial classification of the *C. nervosus* endosymbiont as *Purcelliella* was likely incorrect.

### 3.2 Phylogenetic Relationships of Cixiid Primary Endosymbionts

**FIGURE 2:**
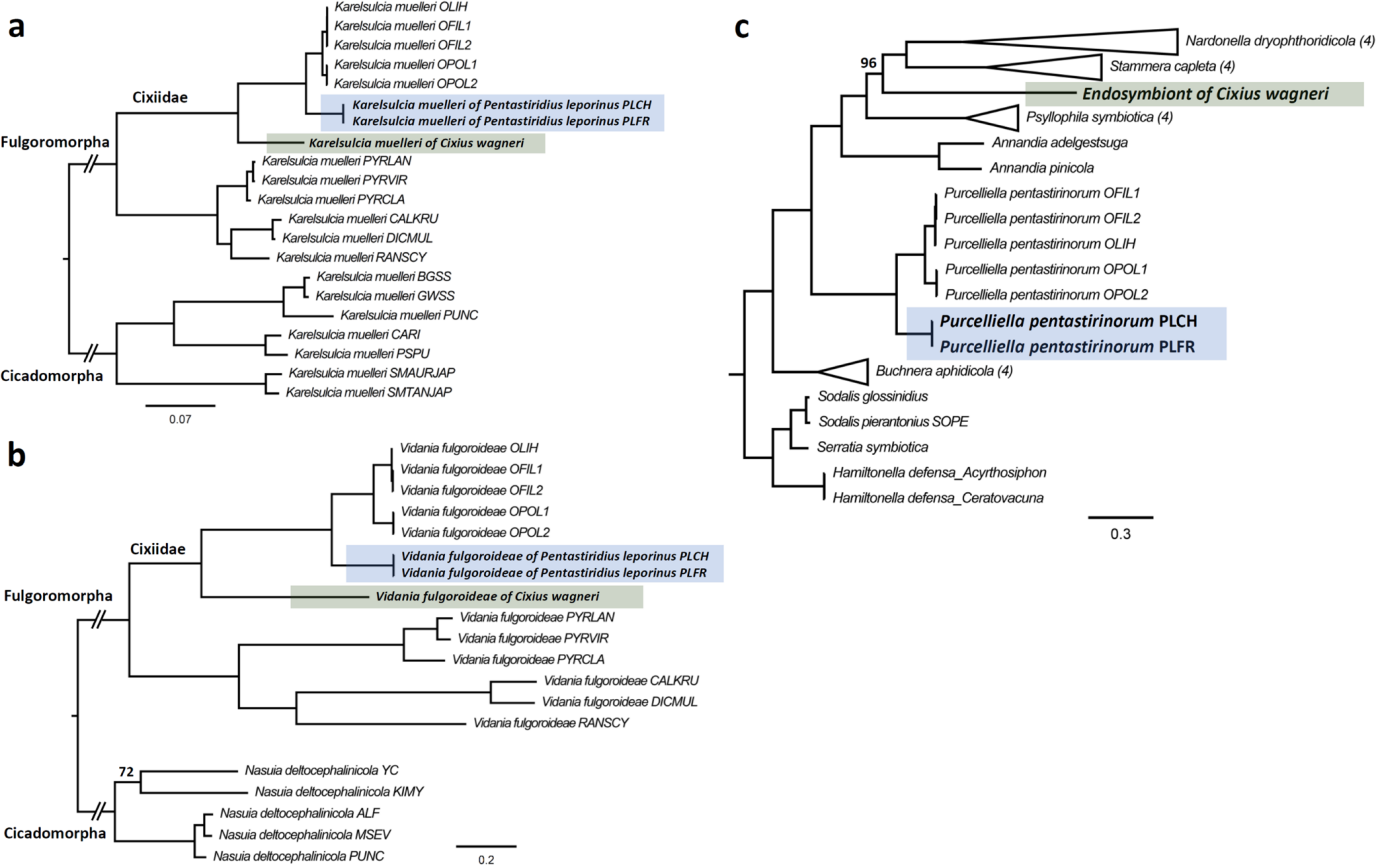
Phylogenetic relationships between Cixiidae endosymbionts. (a, b) Core‐genome maximum‐likelihood trees for *Karelsulcia* (a) and *Vidania* (b) endosymbionts of planthoppers (Fulgoromorpha). *Karelsulcia* and *Nasuia* genomes from Cicadomorpha were used as outgroups. (c) Core‐genome maximum‐likelihood tree of 26 nutritional endosymbionts from the *Gammaproteobacteria*, with *Sodalis* spp., *Serratia symbiotica* and *Hamiltonella defensa* as outgroups. The endosymbionts of *C. wagneri* and *P. leporinus* are colour‐coded based on host species. Branch support is based on 1000 bootstrap iterations. All branches have full bootstrap support unless indicated otherwise.

For *Karelsulcia* and *Vidania*, five genome sequences are currently available from the two Cixiidae species *O. filicicola* and *O. polyphemus* (Bennett and Mao 2018; Gossett et al. 2023). Six additional genomes for each endosymbiont from two other families of the Fulgoromopha, i.e., the Dictyopharidae (strains CALKRU, DICMUL, and RANSCY) and Fulgoridae (strains PYRCLA, PYRLAN, and PYRVIR) (Michalik et al. 2021, 2023) were also included in the analyses. Maximum‐likelihood core‐genome phylogenomic analyses based on 64 conserved single‐copy protein‐coding genes for *Karelsulcia* and 42 genes for *Vidania* (with ‘*Ca*. Nasuia deltocephalinicola’ as outgroup) revealed the same phylogenetic relationships between the endosymbionts of *C. wagneri* , *P. leporinus*, and other Fulgoromorpha: for both endosymbionts, the genomes formed fully supported clades corresponding to the three planthopper families, as expected due to strict host–symbiont co‐evolution. Among the Cixiidae, the endosymbionts of *C. wagneri* were always in the most early‐branching position, followed by the endosymbionts of *P. leporinus* and *Oliarus* spp. (Figure 2a,b). This is also in line with the hosts' phylogenetic relationships, since *Oliarus* sp. and *P. leporinus* belong to the Pentastirinian sublineage within the Cixiidae family, whereas *C. wagneri* is part of the Cixiinian sublineage, a sister clade to the Pentastirinian lineage (Bucher et al. 2023).

Since the host range of *Purcelliella* appears to be restricted to Cixiidae, only five genomes from *O. filicicola* and *O. polyphemus* are currently available. A maximum‐likelihood phylogenomic analysis based on 29 single‐copy protein‐coding genes conserved in 31 nutritional endosymbionts from the *Gammaproteobacteria* showed the same fully supported relationship, with *Purcelliella* from *P. leporinus* in an early‐branching position compared to *Purcelliella* from *Oliarus* spp. (Figure 2c). In contrast, the *Gammaproteobacteria* symbiont from *C. wagneri* was placed in a different position (96% bootstrap support, Figure 2c), as an isolated branch most closely related to nutritional endosymbionts of Sternorrhyncha (‘*Ca*. Annandia’ spp. from adelgids (Dial et al. 2022) and ‘*Ca*. Psyllophila symbiotica’ from psyllids (Dittmer et al. 2023)) and beetles (‘*Ca*. Stammera capleta’ from tortoise leaf beetles (Salem et al. 2020) and ‘*Ca*. Nardonella dryophthoridicola’ from weevils (Anbutsu et al. 2017)). Considering the split genes of the *Gammaproteobacteria* symbiont from *C. wagneri* , the phylogenomic tree was produced with the CDS from the PGAP annotation but also using only complete genes without internal stop codons. Both analyses produced the same tree topology.

An additional phylogenetic analysis of near‐complete 16S rRNA genes, including *Purcelliella* symbionts from other planthoppers and the endosymbiont of *C. nervosus* , also produced the same pattern: As in the core genome phylogeny, the endosymbionts of *C. wagneri* and *C. nervosus* clustered together, forming a species‐level clade distinct from *Purcelliella* (Figure S1). This confirms that the *Cixius* spp. symbionts represent a new genus and species, for which we propose the name ‘*Ca*. Mirabilia symbiotica’, in homage to Paul Buchner who referred to the Auchenorrhyncha as a ‘veritable wonderland of insect symbiosis’ (Buchner 1953) (see species description at the end of the discussion).

### 3.3 Similar Metabolic Complementarity Despite Changing Partners

**FIGURE 3:**
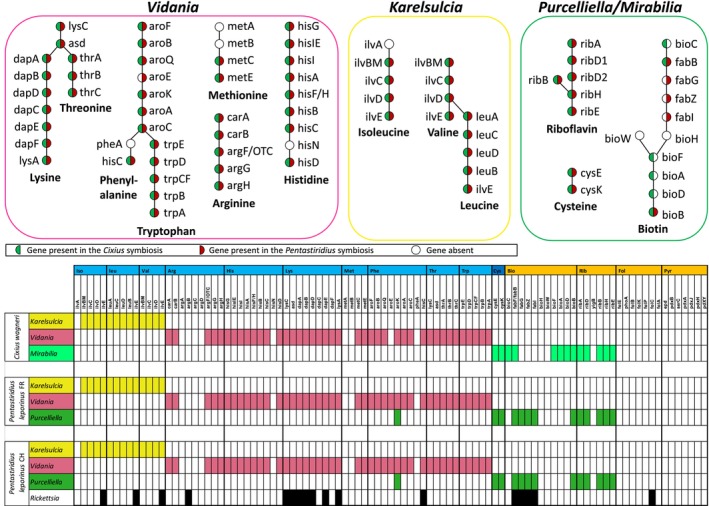
The different endosymbiont consortia retain similar nutritional functions. The upper panel shows the nutritional biosynthesis pathways encoded by each primary endosymbiont. Genes present in the endosymbionts of *C. wagneri* are shown in green, genes present in the endosymbionts of *P. leporinus* in red. Together, the three primary endosymbionts of each host species retain genes involved in the biosynthesis of all essential amino acids, the non‐essential amino acid cysteine and the B vitamins riboflavin and biotin. The lower panel illustrates the metabolic repertoires of all endosymbionts for each of the three sequenced individuals.

We next compared the metabolic complementarity between the 3 co‐primary endosymbionts of *C. wagneri* and *P. leporinus* for the production of the 10 essential amino acids as well as B vitamins (Figure 3). In both species, *Karelsulcia* is only able to synthesise the three branched‐chain amino acids leucine, isoleucine, and valine. The gene coding for *ilvA* (the initial reaction in the isoleucine biosynthesis pathway) is missing, but this is consistent with all *Karelsulcia* genomes from Fulgoromorpha sequenced so far (12 genomes from 9 host species representing four families) (Michalik et al. 2021, 2024; Bennett and Mao 2018; Gossett et al. 2023; Deng et al. 2023). In contrast, *Vidania* encodes genes for the seven other EAAs, with a single difference between the two insect species: the gene *aroE* from the Shikimate pathway is present in *Vidania* from *P. leporinus* but is missing in *Vidania* from *C. wagneri* (Figure 3). Apart from that, *Vidania* from both species encode arginine via the alternative pathway *carAB‐argFGH*, the complete lysine, threonine, and tryptophan pathways, an almost complete histidine pathway (only *hisN* is missing), and the last two reactions of the methionine pathway (*metC*, *metE*) (Figure 3). This functional repertoire is similar to *Vidania* from other Fulgoromorpha, except that the genes *dapC/argD* in the lysine biosynthesis pathway and *aroF/aroG* in the Shikimate pathway are often missing (Michalik et al. 2021, 2024; Gossett et al. 2023; Deng et al. 2023) but are present here in both species.

In contrast to *Karelsulcia* and *Vidania*, *Purcelliella* from *P. leporinus* does not possess genes coding for EAAs but can produce the non‐essential amino acid cysteine (Figure 3), like *Purcelliella* from *Oliarus* spp. (Bennett and Mao 2018; Gossett et al. 2023). In addition, *Purcelliella* encodes riboflavin (except for the gene *yigB*, which is missing in all *Purcelliella* sequenced so far (Gossett et al. 2023)) and the last reaction (*bioB*) of the biotin pathway (Figure 3). Hence, *Purcelliella* from *P. leporinus* has a similar gene set for B vitamins to *Purcelliella* of *O. filicicola*, whereas the biotin pathway is complete in *Purcelliella* of *O. polyphemus* (Gossett et al. 2023). Despite its much smaller genome, the *Gammaproteobacteria* endosymbiont *M. symbiotica* of *C. wagneri* possesses a similar gene set for the cysteine and riboflavin pathways as *Purcelliella*, as well as an almost complete biotin biosynthesis pathway, missing only *bioH* and *bioW* (Figure 3). Among these, *bioA* and *ribD* were split into two parts due to an internal stop codon.

### 3.4 *P. leporinus* Harbours a *Rickettsia* Symbiont From the Meloidae Group

The assemblies of both *P. leporinus* specimens from France and Switzerland also contained numerous contigs belonging to *Rickettsia*, raising the question of whether this common insect symbiont could further complement the metabolic repertoire of the three primary endosymbionts. Despite its low coverage of 18×, the genome of the *Rickettsia* strain from PLCH could be assembled into 107 contigs with a total length of 1.33 Mbp and 1272 protein‐coding genes (Table 1). This is comparable to other recently published *Rickettsia* genomes from diverse insects (Davison et al. 2022), and CheckM indicated high genome completeness (86.76%) and extremely low contamination (0.07%). The *Rickettsia* assembly from PLFR was less complete (1.30 Mbp in 130 scaffolds) but highly similar (99.80% ANI) to the *Rickettsia* from PLCH (Table 1).

**FIGURE 4:**
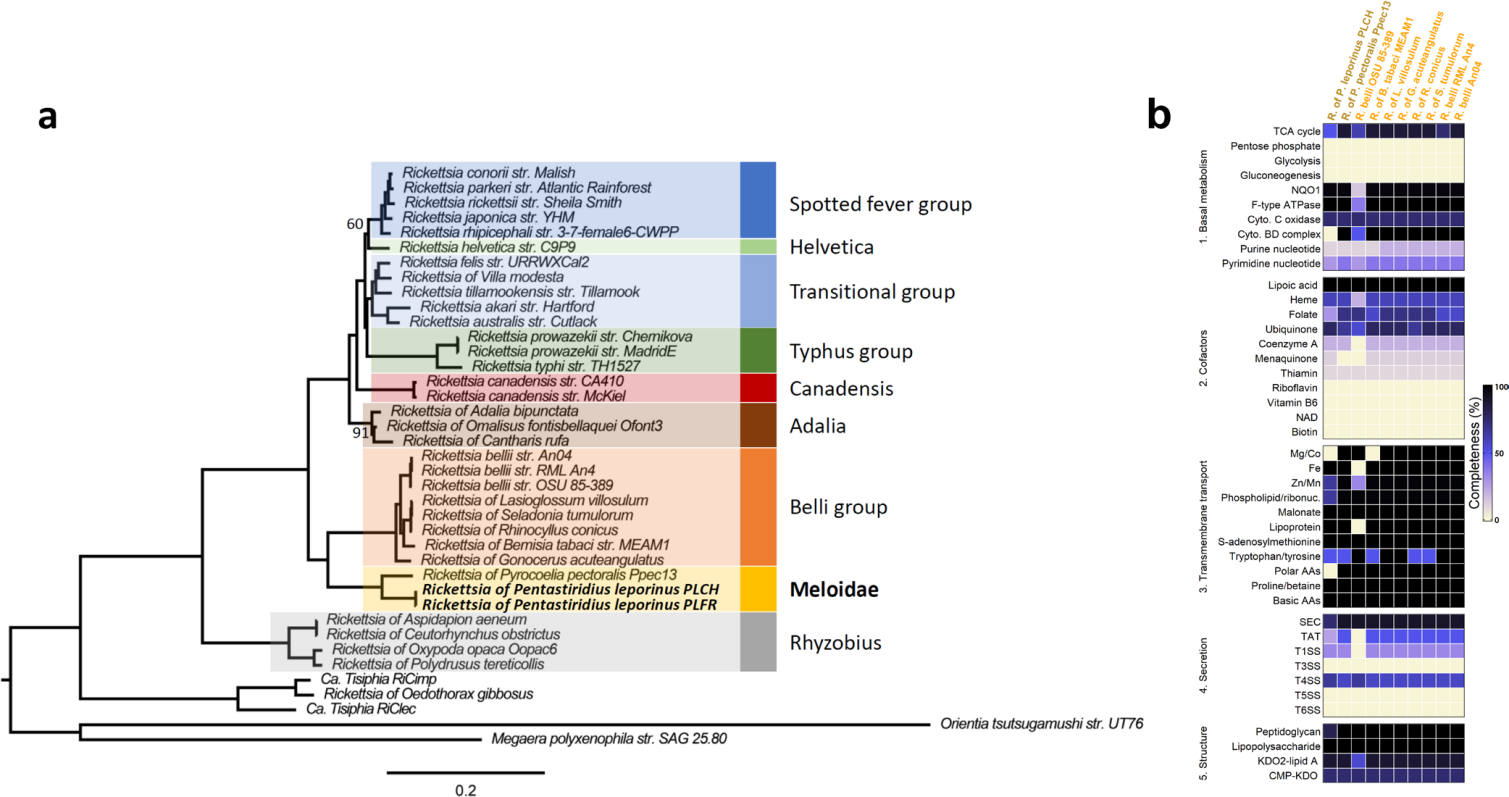
The *Rickettsia* symbiont of *P. leporinus* belongs to the Meloidae group and shows typical hallmarks of *Rickettsia* genomes. (a) Core‐genome maximum‐likelihood tree of 34 *Rickettsia* genomes spanning the genetic diversity of this genus. Genomes from ‘*Ca*. Tisiphia’ spp., ‘*Ca*. Megaera polyxenophila’ strain SAG 25.80 and *Orientia tsutsugamushi* strain UT76 were used as an outgroup. The *Rickettsia* symbionts of *P. leporinus* are highlighted in bold. Branch support is based on 1000 bootstrap iterations. All branches have full bootstrap support unless indicated otherwise. (b) Heatmap showing the biosynthetic capacities of *Rickettsia* strains from the Meloidae and Belli groups based on KEGG pathway annotation. Only the more contiguous *Rickettsia* genome assembly from *P. leporinus* PLCH was included in this analysis. Colours indicate pathway completeness computed by KEGGDecoder. Pathways for EAAs are not shown as these are already presented in Figure 3. CMP‐KDO, cytidine 5′‐monophospho‐3‐deoxy‐d‐manno‐2‐octulosonic acid; LPS, lipopolysaccharide; NAD, nicotinamide adenine dinucleotide; NQO, NADH quinone oxidoreductase; Sec, general secretion; SS, secretion system; TAT, twin arginine translocation.

A phylogenomic analysis based on 240 single‐copy protein‐coding genes conserved in 34 *Rickettsia* genomes representing the genetic diversity of this genus revealed that the *Rickettsia* strains from *P. leporinus* clustered with full bootstrap support together with the *Rickettsia* strain of the firefly *Pyrocoelia pectoralis* (Coleoptera), the only available genome for the recently proposed Meloidae group (Davison et al. 2022) (Figure 4a). The Meloidae group is a sister group to the Belli group, which also encompasses *Rickettsia* strains associated with diverse insects such as bees, whiteflies, and weevils as well as ticks (Figure 4a).

In terms of metabolic capabilities, the more contiguous genome of the new *Rickettsia* strain PLCH is very similar to the Meloidae strain of *P. pectoralis* and to several strains from the Belli group (Figure 4b). It features typical hallmarks of *Rickettsia* genomes, such as extremely limited metabolic capabilities and dependence on the host for carbon sources and metabolic precursors (Davison et al. 2022; Salje 2021; Castelli et al. 2024). Hence, the glycolysis, gluconeogenesis, and pentose‐phosphate pathways are completely absent in the *Rickettsia* strain from PLCH and the bacterium cannot synthesise nucleotides (Figure 4b). Concerning EAAs, only the pathway for lysine is almost complete, with only two missing genes (*dapC* and *lysA*) (Figure 3). Vitamins and co‐factors are also scarce, only the biosynthesis pathway for lipoic acid is complete and the one for ubiquinol is almost complete (Figure 4b). However, considering the fragmented state of this genome assembly, it is possible that some genes were missed in our assembly and that some of these pathways are actually functional. To scavenge ATP, precursors, and nucleotides directly from the host environment, the genome encodes numerous transporters (Figure 4b), including various ABC and *tlc* transporters, notably ATP/ADP exchange transporters and nucleotide exchange transporters. In addition, the bacterium possesses a type IV secretion system (Figure 4b), several patatin‐like phospholipases as well as numerous ankyrin‐ and tetratricopeptide‐repeat‐containing proteins, which have all been implicated in the interaction between *Rickettsia* and their host's cells (Salje 2021; Castelli et al. 2024).

## 4 Discussion

The Auchenorrhyncha (cicadas, leafhoppers, spittlebugs, and planthoppers) are renowned for their nutritional endosymbiont consortia that have co‐evolved with their insect hosts for tens or even hundreds of millions of years (Michalik et al. 2021, 2023; Koga et al. 2013; McCutcheon et al. 2009; Bennett and Mao 2018; Deng et al. 2023). Herein, we assembled the genomes of the tripartite endosymbiont consortia of the two planthopper species *C. wagneri* and *P. leporinus* from the Cixiidae family. These are the first endosymbiont genomes for this planthopper family apart from those of the Hawaiian species *O. filicicola* and *O. polyphemus* (Bennett and Mao 2018; Gossett et al. 2023).

*P. leporinus* harboured the three primary endosymbionts ‘*Ca*. Karelsulcia muelleri’, ‘*Ca*. Vidania fulgoroideae’, and ‘*Ca*. Purcelliella pentastirinorum’. This tripartite endosymbiont consortium was expected, as it had been observed in all other Cixiidae species investigated so far (Michalik et al. 2023; Bennett and Mao 2018; Gossett et al. 2023; Gonella et al. 2011; Bressan, Arneodo, et al. 2009), and *Purcelliella* had even been originally described in *P. leporinus* planthoppers from eastern France (Bressan, Arneodo, et al. 2009). The *P. leporinus* endosymbionts are almost identical in size, gene content and synteny to the corresponding endosymbionts from *O. filicicola* OLIH, except for a large inversion between the *Karelsulcia* genomes of *P. leporinus* and *O. filicicola* OLIH. The latter is almost identical to the inversion between *Karelsulcia* from diverse planthoppers including *Oliarus* spp. and *Karelsulcia* from leafhoppers (Bennett and Mao 2018; Deng et al. 2023). In terms of biosynthetic capabilities, *Karelsulcia* and *Vidania* from *P. leporinus* jointly encode all 10 essential amino acids, as previously reported in other planthoppers (Michalik et al. 2021; Bennett and Mao 2018; Gossett et al. 2023; Deng et al. 2023). Specifically, *Karelsulcia* possesses all necessary genes (except *ilvA*) for the three branched‐chain EAAs, whereas *Vidania* encodes complete or almost complete gene sets for the other seven EAAs. Despite its larger genome, *Purcelliella* from *P. leporinus* does not possess genes coding for EAAs but encodes the non‐essential amino acid cysteine as well as riboflavin. Other vitamins and co‐factors are absent, except for the last reaction of the biotin pathway. This metabolic repertoire is similar to *Purcelliella* from *O. filicicola*, but greatly reduced compared to *Purcelliella* from *O. polyphemus* , which retains complete biosynthesis pathways for biotin, riboflavin and pyridoxine (except *pdxH*) (Bennett and Mao 2018; Gossett et al. 2023). These different metabolic repertoires may be the result of different selection pressures acting on the endosymbiont depending on the nutrients present in the phloem sap of the host plants of different planthopper species (Gossett et al. 2023).

Interestingly, *C. wagneri* harboured a different and yet unknown Gammaproteobacterium in addition to *Karelsulcia* and *Vidania* for which we propose the name ‘*Ca*. Mirabilia symbiotica’. Its genome has the typical hallmarks of a long endosymbiotic association, i.e., extremely small genome size, low GC content, and reduced metabolic capabilities, suggesting that *Mirabilia* is the third primary endosymbiont of *C. wagneri* , analogous to *Purcelliella* in *P. leporinus* . In addition, 16S rRNA sequence analysis indicated that *C. nervosus* harbours a symbiont belonging to the same species. This is intriguing in light of Müller's observations of a different endosymbiont accompanying *Karelsulcia* and *Vidania* in *C. nervosus* (called ‘b symbiont’) compared to *Oliarus villosus* (‘c + d symbiont’, a.k.a. *Purcelliella*) (Buchner 1953). Müller further documented different bacteriome organisations in the two Cixiidae species: in *C. nervosus* , the bacteriomes harbouring the ‘b symbiont’ were surrounded by bacteriomes harbouring *Karelsulcia*, whereas *Purcelliella* bacteriomes are surrounded by bacteriomes containing *Vidania* (Michalik et al. 2023; Bressan and Mulligan 2013). Unfortunately, we were not able to investigate the precise bacteriome localisation of *Mirabilia* for this study to confirm whether it corresponds to the ‘b symbiont’, since all specimens collected for this study were used for DNA extraction and none were appropriately preserved for microscopy.

Considering its much smaller genome, *Mirabilia* is likely a more ancient symbiont than *Purcelliella*, and a broader sampling of Cixiidae species is needed to understand the distribution of the two endosymbionts across this highly diverse insect family. To date, endosymbionts have been characterised in 12 Cixiidae species, 10 of which belong to the Pentastirinian lineage according to Bucher et al. 2023 and harbour *Purcelliella* (Figure S1). In contrast, the two species harbouring *Mirabilia* ( *C. nervosus* and *C. wagneri* ) belong to the Cixiinian lineage. Hence, it is possible that different infra‐family lineages acquired different endosymbionts throughout their evolution, but additional species from the Cixiinian lineage need to be investigated to consolidate this pattern. Additional *Mirabilia* genomes would also be necessary to validate (or invalidate) the peculiar internal stop codons observed in this work. An alternative hypothesis would be that the ancient symbiont *Mirabilia* was replaced by *Purcelliella* in an ancestor of the Pentastirinian lineage. To fully understand these evolutionary dynamics, it will be necessary to also investigate species from the most basal lineage (Oeclinian lineage), whose endosymbionts have not been characterised to date (Figure S1).

Despite its much smaller genome, *Mirabilia* possesses similar gene sets for EAA and B vitamin biosynthesis as *Purcelliella*, namely complete cysteine and riboflavin pathways as well as an almost complete biotin biosynthesis pathway. This confirms the functional stability of multipartite planthopper endosymbiont consortia despite changing partners over evolutionary time. Interestingly, the same two B vitamins are retained in the genomes of the primary endosymbionts of other phloem sap‐feeding hemipterans, e.g., the psyllid endosymbionts ‘*Ca*. Psyllophila symbiotica’ and ‘*Ca*. Profftella armatura’ (Dittmer et al. 2023; Nakabachi et al. 2020), highlighting the importance of these B vitamins for insects sharing this diet.

Considering the highly reduced genomes and poor biosynthetic capabilities of both *Mirabilia* and *Purcelliella*, it is conceivable that they could be complemented or replaced in the future by other, more functionally versatile symbionts to meet the nutritional requirements of their insect hosts. Their replacements could be recruited among other facultative insect symbionts, as already observed for *Serratia symbiotica* , *Arsenophonus*, and *Wolbachia* in aphids (Monnin et al. 2020; Manzano‐Marin et al. 2023; Yorimoto et al. 2022; De Clerck et al. 2015), *Sodalis* in spittlebugs (Koga et al. 2013; Koga and Moran 2014), and even for the plant pathogen ‘*Ca*. Liberibacter psyllaurous’ in psyllids (Kwak et al. 2025). *Wolbachia* has indeed been detected in several planthopper species, colonising various tissues such as the ovaries, salivary glands, the gut, fat body, and even sharing bacteriocytes with *Karelsulcia* or *Vidania* (Michalik et al. 2023). Moreover, metagenomic data indicate that at least one of these planthopper‐associated *Wolbachia* strains encodes a complete biotin biosynthesis pathway (Michalik et al. 2021). While we detected *Wolbachia* reads in both *C. wagneri* and *P. leporinus* , it was not possible to assemble these genomes to investigate their metabolic repertoires, and nothing is known regarding the prevalence of *Wolbachia* in natural populations. Interestingly, bacteria resembling *Wolbachia* have been observed in the nuclei of various tissues in *P. leporinus* , but their identity needs to be confirmed using additional methods such as FISH (Arneodo et al. 2008). In addition, *C. wagneri* and *P. leporinus* are known vectors of phytopathogenic *Arsenophonus* strains, which also possess complete biosynthetic pathways for several B vitamins, including biotin, folate, heme, riboflavin, and ubiquinone (Mahillon et al. 2025). However, they are not reliably vertically transmitted and have so far not been observed to colonise the bacteriomes (Bressan, Semetey, et al. 2009; Bressan et al. 2012), making them unlikely candidates as nutritional symbionts.

Finally, both *P. leporinus* individuals sequenced herein harboured a *Rickettsia* symbiont belonging to the recently proposed Meloidae group, so far only observed in the firefly *Pyrocoelia pectoralis* (Coleoptera) (Davison et al. 2022). Our draft genome of the *Rickettsia* symbiont of *P. leporinus* shows no metabolic overlap with the primary endosymbionts due to its overall scarce biosynthetic capabilities. *Rickettsia* symbionts have already been observed in different planthopper species (Michalik et al. 2023), but no genome sequences are currently available. When present, *Rickettsia* were always observed in the cytoplasm of fat body cells and, in the species *Orosanga japonica* (family Ricanidae), also in the nuclei of fat body cells (Michalik et al. 2023). *Rickettsia* transmission between sap‐feeding insects can be vertical but also horizontal via plants, as observed for two strains from the Belli group harboured by the leafhopper *Empoasca papayae* and the whitefly *Bemisia tabaci* (Caspi‐Fluger et al. 2012; Shi et al. 2024; Davis et al. 1998). In contrast to the tissue localisation observed in planthoppers, the *Rickettsia* symbiont of *B. tabaci* colonises a wide range of insect organs, notably the salivary glands and the stylet (Brumin et al. 2012). Moreover, once transmitted into the phloem, it modulates the plant's defences to the benefit of the whitefly and other herbivorous insects (Shi et al. 2024). Hence, a more detailed investigation of the tissular and cellular localisation of the *Rickettsia* associated with *P. leporinus* as well as its potential transmission to plants will be an interesting perspective for future research.

In conclusion, this work identified an ancient nutritional endosymbiont of *C. wagneri* and a *Rickettsia* symbiont associated with *P. leporinus* , highlighting that we are far from having elucidated the diversity, metabolic repertoires and evolutionary dynamics of bacterial endosymbionts associated with planthoppers, an extremely divers insect group.

### 4.1 Description of ‘*Candidatus* Mirabilia symbiotica’ Gen. Nov. sp. Nov

Mi.ra.bi'li.a. sym.bio'ti.ca. N. L. fem. n. Etymology: N. L. masc/fem. adj. *mirabilis*, ‘wonderful, marvellous’; N. L. fem. adj. *symbiotica*, ‘living together’). The name was chosen in homage to Paul Buchner who referred to the Auchenorrhyncha as a ‘veritable wonderland of insect symbiosis’.

*Gammaproteobacteria* endosymbiont of the planthopper *C. wagneri*. Basis of assignment: 16S rRNA gene sequence and complete genome sequence (Bioproject accession: PRJNA1100464).

## Author Contributions

**Jessica Dittmer:** conceptualization, investigation, funding acquisition, writing – original draft, formal analysis, data curation. **Mathieu Mahillon:** conceptualization, investigation, writing – review and editing, formal analysis, data curation. **Christophe Debonneville:** writing – review and editing, investigation. **Franco Faoro:** funding acquisition, writing – review and editing, supervision. **Xavier Foissac:** investigation, funding acquisition, writing – review and editing, supervision. **Olivier Schumpp:** funding acquisition, writing – review and editing, supervision. **Bessem Chouaia:** conceptualization, writing – review and editing, formal analysis.

## Conflicts of Interest

The authors declare no conflicts of interest.

## Supporting information


**Table S1:** List of insect endosymbiont genomes included in the phylogenomic analyses.
**Table S2:** Genome annotation of the new *Gammaproteobacteria* endosymbiont of *Cixius wagneri* after manually merging split genes.
**Figure S1:** Intersection plots showing the amount of shared orthogroups between the endosymbionts of *Cixius wagneri* and *Pentastiridius leporinus* with the corresponding endosymbionts from *Oliarus* spp. (i.e., strains OLIH, OFIL1, OFIL2, OPOL1, OPOL2) and from other Fulgoromorpha (i.e., the strains CALKRU, DICMUL, RANSCY, PYRCLA, PYRLAN, PYRVIR).
**Figure S2:** Maximum‐likelihood phylogenetic tree based on the 16S rRNA genes of 41 nutritional endosymbionts from the *Gammaproteobacteria*, with *Sodalis* spp., *Serratia symbiotica* and *Hamiltonella defensa* as outgroup. Colours indicate different genera. The endosymbionts of *C. wagneri* and *C. nervosus* cluster together and represent a new species‐level clade. Branch support is based on 1000 bootstrap iterations.
**Figure S3:** Schematic representation of *Gammaproteobacteria* endosymbiont distribution across all Cixiidae species investigated to date. Subfamily lineages are based on Bucher et al. (2023). For each lineage, the investigated host species and their *Gammaproteobacteria* endosymbionts are shown. Endosymbiont data is based on Michalik et al. (2023), Bennett and Mao (2018), Gossett et al. (2023), and Bressan, Arneodo, et al. (2009) and this study.

## Data Availability

The genomes produced in this work have been deposited in the NCBI database and will be made available upon publication under BioProject accessions PRJNA1100464 (endosymbionts of Cixius wagneri) and PRJNA1100466 (endosymbionts of Pentastiridius leporinus).
