# Supplementary material for: The Endosymbiont Consortia of Two Cixiidae Planthoppers Reveal an Ancient Symbiosis With ‘Candidatus Mirabilia Symbiotica’
Source: Environ Microbiol Rep. 2025 Oct 8;17(5):e70204. doi: 10.1111/1758-2229.70204 (PMC12505427; doi:10.1111/1758-2229.70204)
Supplement: Supplementary file 1 — Table S1: List of insect endosymbiont genomes included in the phylogenomic analyses. Table S2: Genome annotation of the new Gammaproteobacteria endosymbiont of Cixius wagneri after manually merging split genes. Figure S1: Intersection plots showing the amount of shared orthogroups between the endosymbionts of Cixius wagneri and Pentastiridius leporinus with the corresponding endosymbionts from Oliarus spp. (i.e., strains OLIH, OFIL1, OFIL2, OPOL1, OPOL2) and from other Fulgoromorpha (i.e., the strains CALKRU, DICMUL, RANSCY, PYRCLA, PYRLAN, PYRVIR). Figure S2: Maximum‐likelihood phylogenetic tree based on the 16S rRNA genes of 41 nutritional endosymbionts from the Gammaproteobacteria, with Sodalis spp., Serratia symbiotica and Hamiltonella defensa as outgroup. Colours indicate different genera. The endosymbionts of C. wagneri and C. nervosus cluster together and represent a new species‐level clade. Branch support is based on 1000 bootstrap iterations. Figure S3: Schematic representation of Gammaproteobacteria endosymbiont distribution across all Cixiidae species investigated to date. Subfamily lineages are based on Bucher et al. (2023). For each lineage, the investigated host species and their Gammaproteobacteria endosymbionts are shown. Endosymbiont data is based on Michalik et al. (2023), Bennett and Mao (2018), Gossett et al. (2023), and Bressan, Arneodo, et al. (2009) and this study. [file EMI4-17-e70204-s001.pdf]

SUPPLEMENTARY MATERIAL

The endosymbiont consortia of two Cixiidae planthoppers reveal an ancient symbiosis with '*Candidatus* Mirabilia symbiotica'

Jessica Dittmer[a,b]#, Mathieu Mahillon[c,d]*, Christophe Debonneville[c], Franco Faoro[a], Xavier Foissac[e], Olivier Schumpp[c], Bessem Chouaia[e]

[a]Dipartimento di Scienze agrarie e ambientali, Università degli Studi di Milano, Italy
[b]UMR 1345, Université d'Angers, Institut Agro, INRAE, IRHS, SFR Quasav, Angers, France
[c]Research group Virology, Bacteriology and Phytoplasmology, Agroscope, Nyon, Switzerland
[d]Department of Plants and Crops, Faculty of Bioscience Engineering, Ghent University
[e]UMR 1332 Biologie du Fruit et Pathologie, Université de Bordeaux, INRAE, Bordeaux, France

Supplementary Table S1. List of insect endosymbiont genomes included in the phylogenomic analyses.

Bacterium	Host species	Accession
Ca. Karelsulcia muelleri CALKRU	*Callodictya krueperi*	GCF_016889585.1
Ca. Karelsulcia muelleri DICMUL	*Dictyophara multireticulata*	GCF_016888765.1
Ca. Karelsulcia muelleri OFIL1	*Oliarus filicicola*	GCF_028748865.1
Ca. Karelsulcia muelleri OFIL2	*Oliarus filicicola*	GCF_028856225.1
Ca. Karelsulcia muelleri OLIH	*Oliarus filicicola*	GCF_003391295.1
Ca. Karelsulcia muelleri OPOL1	*Oliarus polyphemus*	GCF_028856205.1
Ca. Karelsulcia muelleri OPOL2	*Oliarus polyphemus*	GCF_028673255.1
Ca. Karelsulcia muelleri PYRCLA	*Pyrops clavatus*	GCF_022985555.1
Ca. Karelsulcia muelleri PYRLAN	*Pyrops lathburii*	GCF_022985515.1
Ca. Karelsulcia muelleri PYRVIR	*Pyrops viridirostris*	GCF_022985615.1
Ca. Karelsulcia muelleri RANSCY	*Ranissus scytha*	GCF_016888785.1
Ca. Karelsulcia muelleri BGSS	*Graphocephala atropunctata*	GCF_000754305.1
Ca. Karelsulcia muelleri CARI	*Clastoptera arizonana*	GCF_000147035.1
Ca. Karelsulcia muelleri GWSS	*Homalodisca vitripennis*	GCF_000017525.1
Ca. Karelsulcia muelleri PSPU	*Philaenus spumarius*	GCF_000829155.1
Ca. Karelsulcia muelleri PUNC	*Macrosteles quadripunctulatus*	GCF_001447915.1
Ca. Karelsulcia muelleri SMAURJAP	*Auritibicen japonicus*	GCF_003213655.1
Ca. Karelsulcia muelleri SMTANJAP	*Tanna japonensis*	GCF_003215265.1
Ca. Vidania fulgoroideae CALKRU	*Callodictya krueperi*	GCA_017348935.1
Ca. Vidania fulgoroideae DICMUL	*Dictyophara multireticulata*	GCA_017348955.1
Ca. Vidania fulgoroideae OFIL1	*Oliarus filicicola*	GCA_028748845.1
Ca. Vidania fulgoroideae OFIL2	*Oliarus filicicola*	GCA_028856185.1
Ca. Vidania fulgoroideae OLIH	*Oliarus filicicola*	GCA_003391315.1
Ca. Vidania fulgoroideae OPOL1	*Oliarus polyphemus*	GCA_028856065.1
Ca. Vidania fulgoroideae OPOL2	*Oliarus polyphemus*	GCA_028748825.1
Ca. Vidania fulgoroideae PYRCLA	*Pyrops clavatus*	GCA_022985575.1
Ca. Vidania fulgoroideae PYRLAN	*Pyrops lathburii*	GCA_022985595.1
Ca. Vidania fulgoroideae PYRVIR	*Pyrops viridirostris*	GCA_022985535.1
Ca. Vidania fulgoroideae RANSCY	*Ranissus scytha*	GCA_017348975.1
Ca. Nasuia deltocephalinicola ALF	*Macrosteles quadrilineatus*	GCA_000442605.1
Ca. Nasuia deltocephalinicola KIMY	*Nesophrosyne* sp.	GCA_024818655.1
Ca. Nasuia deltocephalinicola MSEV	*Macrosteles severini*	GCA_014211875.1
Ca. Nasuia deltocephalinicola PUNC	*Macrosteles quadripunctulatus*	GCA_001447885.1
Ca. Nasuia deltocephalinicola YC	Unknown	GCA_017086605.1
Ca. Annandia adelgestsuga	*Adelges tsugae*	GCF_003956045.1
Ca. Annandia pinicola	*Pineus similis*	GCF_020541245.1
Buchnera aphidicola APS	*Acyrthosiphon pisum*	GCF_000009605.1
Buchnera aphidicola	*Aphis craccivora*	GCF_013487785.1
Buchnera aphidicola	*Myzus persicae*	GCA_026684135.1
Buchnera aphidicola	*Schizaphis graminum*	GCF_003099975.1
Ca. Hamiltonella defensa 5AT	*Acyrthosiphon pisum*	GCF_000021705.1
Ca. Hamiltonella defensa	*Ceratovacuna japonica*	GCA_024349745.1

Ca. Nardonella dryophthoridicola Epo	*Euscepes postfasciatus*	GCF_004296535.1
Ca. Nardonella dryophthoridicola Pin	*Pachyrhynchus infernalis*	GCF_004296515.1
Ca. Nardonella dryophthoridicola NardRF	*Rhynchophorus ferrugineus*	GCF_017656055.1
Ca. Nardonella dryophthoridicola Sgi	*Sipalinus gigas*	GCF_004296475.1
Ca. Psyllophila symbiotica PSmelAO1	*Cacopsylla melanoneura*	GCA_030849185.1
Ca. Psyllophila symbiotica PSpicST1	*Cacopsylla picta*	GCA_030849285.1
Ca. Psyllophila symbiotica PSpyr	*Cacopsylla pyri*	GCA_030849405.1
Ca. Psyllophila symbiotica PSpyc	*Cacopsylla pyricola*	GCA_030849345.1
Ca. Purcelliella pentastirinorum OFIL1	*Oliarus filicicola*	GCF_028748805.1
Ca. Purcelliella pentastirinorum OFIL2	*Oliarus filicicola*	GCF_028856425.1
Ca. Purcelliella pentastirinorum OLIH	*Oliarus filicicola*	GCF_003391335.1
Ca. Purcelliella pentastirinorum OPOL1	*Oliarus polyphemus*	GCF_028856405.1
Ca. Purcelliella pentastirinorum OPOL2	*Oliarus polyphemus*	GCF_028748785.1
Serratia symbiotica	*Aphis fabae*	GCF_009831665.3
Sodalis glossinidius	*Glossina morsitans morsitans*	GCF_000010085.1
Ca. Sodalis pierantonius SOPE	*Sitophilus oryzae*	GCF_000517405.1
Ca. Stammera capleta	*Cassida rubiginosa*	GCA_002688505.1
Ca. Stammera capleta	*Charidotella sexpunctata*	GCA_015139955.1
Ca. Stammera capleta	*Chelymorpha alternans*	GCA_015134415.1
Ca. Stammera capleta	*Ischnocodia annulus*	GCA_015134395.1
Rickettsia akari str. Hartford	Mite-borne human pathogen	GCF_000018205.1
Rickettsia australis str. Cutlack	Tick-borne human pathogen	GCF_000284155.1
Rickettsia bellii str. An04	*Amblyomma neumanni*	GCF_002078315.1
Rickettsia bellii str. OSU 85-389	*Dermacentor variabilis*	GCF_000018245.1
Rickettsia bellii str. RML An4	*Amblyomma neumanni*	GCF_000965005.1
Rickettsia canadensis str. CA410	Human pathogen with unknown vector	GCF_000283915.1
Rickettsia canadensis str. McKiel	Human pathogen with unknown vector	GCF_000014345.1
Rickettsia conorii str. Malish	Tick-borne human pathogen	GCF_000007025.1
Rickettsia felis str. URRWXCal2	Flea-borne human pathogen	GCA_000012145.1
Rickettsia helvetica str. C9P9	Tick-borne human pathogen	GCF_000255355.1
Rickettsia japonica str. YHM	Tick-borne human pathogen	GCF_002356695.1
Rickettsia parkeri str. Atlantic Rainforest	Tick-borne human pathogen	GCF_005549115.1
Rickettsia prowazekii str. Chernikova	Louse-borne human pathogen	GCF_000277165.1
Rickettsia prowazekii str. MadridE	Louse-borne human pathogen	GCF_000195735.1
Rickettsia rhipicephali str. 3-7-female6-CWPP	Tick-borne human pathogen	GCF_000284075.1
Rickettsia rickettsii str. Sheila Smith	Tick-borne human pathogen	GCF_000018225.1
Rickettsia tillamookensis str. Tillamook	*Ixodes pacificus*	GCF_016743795.2
Rickettsia typhi str. TH1527	Flea-borne human pathogen	GCF_000277285.1
Rickettsia sp.	*Adalia bipunctata*	dx.doi.org/10.5061/dryad.6cn66
Rickettsia sp.	*Aspidapion aeneum*	GCF_964030775.1
Rickettsia sp. str. MEAM1	*Bemisia tabaci*	GCF_002285905.1
Rickettsia sp.	*Cantharis rufa*	GCF_964026445.1
Rickettsia sp.	*Ceutorhynchus obstrictus*	GCF_964026565.1
Rickettsia sp.	*Gonocerus acuteangulatus*	GCF_964026435.1

Rickettsia sp.	*Lasioglossum villosulum*	GCF_964026455.1
Rickettsia sp.	*Oedothorax gibbosus*	GCF_936269705.1
Rickettsia sp. str. Ofont3	*Omalisus fontisbellaquei*	GCA_020404465.1
Rickettsia sp. str. Oopac6	*Oxypoda opaca*	GCA_020881235.1
Rickettsia sp.	*Polydrusus tereticollis*	GCF_964026385.1
Rickettsia sp. str. Ppec13	*Pyrocoelia pectoralis*	GCA_020404425.1
Rickettsia sp.	*Rhinocyllus conicus*	GCF_964026465.1
Rickettsia sp.	*Seladonia tumulorum*	GCF_964030815.1
Rickettsia sp.	*Villa modesta*	GCA_964026485.1
Ca. Megaera polyxenophila str. SAG 25.80	*Cryptomonas gyropyrenoidosa*	GCF_029982035.1
Orientia tsutsugamushi UT76	Mite-borne human pathogen	GCF_900327255.1
Ca. Tisiphia str. RiCimp	*Culicoides impunctatus*	GCA_020410785.1
Ca. Tisiphia str. RiClec	*Cimex lectularius*	GCA_020410805.1

Supplementary Table S2. Genome annotation of the new *Gammaproteobacteria* endosymbiont of *Cixius wagneri* after manually merging split genes.

ID	Type	Start	End	Strand	GeneID	Product
CW_00001	CDS	1	840	+	dnaX	DNA polymerase III subunit gamma/tau
CW_00002	CDS	949	1095	+		hypothetical protein
CW_00003	CDS	1097	1555	+		hypothetical protein
CW_00004	CDS	1564	3261	+	htpG	molecular chaperone htpG
CW_00005	CDS	3502	3984	-	bioD	dethiobiotin synthase bioD
CW_00006	CDS	4037	4717	-	bioC	malonyl-ACP O-methyltransferase BioC
CW_00007	CDS	4727	5728	-	bioF	8-amino-7-oxononanoate synthase bioF
CW_00008	CDS	5865	6782	-	bioB	biotin synthase BioB
CW_00009	CDS	6823	7944	+	bioA	adenosylmethionine--8-amino-7-oxononanoate transaminase bioA
CW_00010	CDS	8093	9295	+	tyrS	tyrosine--tRNA ligase
CW_00011	CDS	9355	9744	-	iscU	Fe-S cluster assembly scaffold IscU
CW_00012	CDS	9783	10943	-	iscS	cysteine desulfurase IscS
CW_00013	CDS	11022	11543	+	ribA	GTP cyclohydrolase II
CW_00014	CDS	11546	11707	-	rpmG	50S ribosomal protein L33
CW_00015	CDS	11736	11957	-	rpmB	50S ribosomal protein L28
CW_00016	CDS	11961	13445	-	proS	proline--tRNA ligase
CW_00017	CDS	13488	14465	+	ribD	riboflavin biosynthesis protein RibD
CW_00018	CDS	14569	15045	+	ribH	6,7-dimethyl-8-ribityllumazine synthase
CW_00019	CDS	15376	15585	+	cspE	transcription antiterminator/RNA stability regulator CspE
CW_00020	CDS	15770	16177	+	rplM	50S ribosomal protein L13
CW_00021	CDS	16178	16573	+	rpsI	30S ribosomal protein S9
CW_00022	CDS	16906	17472	-	tilS	tRNA lysidine(34) synthetase TilS
CW_00023	CDS	17507	19894	-	gyrB	DNA topoisomerase subunit B
CW_00024	CDS	19896	21008	-	dnaN	DNA polymerase III subunit beta
CW_00025	CDS	21034	21177	+	rpmH	50S ribosomal protein L34
CW_00026	CDS	21335	22870	+	yidC	membrane protein insertase YidC
CW_00027	CDS	22923	24284	+	mnmE	tRNA uridine-5-carboxymethylaminomethyl(34) synthesis GTPase MnmE
CW_00028	tRNA	24736	24808	+		tRNA-Asn
CW_00029	tRNA	24842	24929	-		tRNA-Leu
CW_00030	tRNA	25115	25187	-		tRNA-Gly
CW_00031	CDS	25257	26465	-	clpX	ATP-dependent Clp protease ATP-binding subunit ClpX
CW_00032	CDS	26824	27444	-	clpP	ATP-dependent Clp protease proteolytic subunit ClpP
CW_00033	CDS	27620	29002	+	asnS	asparagine--tRNA ligase
CW_00034	CDS	29067	30563	+	gyrA	DNA topoisomerase subunit A
CW_00035	tRNA	30588	30659	+		tRNA-Asp
CW_00036	tRNA	30693	30763	+		tRNA-Trp
CW_00037	CDS	30769	31200	-		hypothetical protein
CW_00038	CDS	31348	32148	-	gltX	glutamate--tRNA ligase
CW_00039	tRNA	32160	32232	+		tRNA-Val

CW_00040	tRNA	32246	32321	+		tRNA-Lys
CW_00041	CDS	32347	33288	+	cysK	cysteine synthase A
CW_00042	CDS	33292	33936	-	ribB	3,4-dihydroxy-2-butanone 4-phosphate synthase
CW_00043	CDS	33943	34251	-	erpA	iron-sulfur cluster insertion protein ErpA
CW_00044	CDS	34280	35137	+	rsmH	16S rRNA (cytosine(1402)-N(4))-methyltransferase RsmH
CW_00045	CDS	35145	37493	+	secA	preprotein translocase subunit SecA
CW_00046	rRNA	37497	37612	-		5S ribosomal RNA
CW_00047	rRNA	37709	40627	-		23S ribosomal RNA
CW_00048	tRNA	40641	40713	-		tRNA-Ala
CW_00049	tRNA	40729	40803	-		tRNA-Ile
CW_00050	rRNA	40870	42451	-		16S ribosomal RNA
CW_00051	CDS	42690	44126	-	rpsA	30S ribosomal protein S1
CW_00052	CDS	44169	47771	-	putA	Trifunctional transcriptional regulator/proline dehydrogenase/pyrroline-5-carboxylate dehydrogenase
CW_00053	CDS	48005	48427	-	rsmD	16S rRNA m(2)G966-methyltransferase
CW_00054	CDS	48562	49272	-	cysE	serine acetyltransferase cysE
CW_00055	CDS	49306	50223	-	rluA	RluA family pseudouridine synthase
CW_00056	CDS	50229	51146	+	rluC	23S rRNA pseudouridine(955/2504/2580) synthase RluC
CW_00057	CDS	51168	51362	+	rpmF	50S ribosomal protein L32
CW_00058	CDS	51364	51594	+	acpP	acyl carrier protein
CW_00059	CDS	52566	56642	-	rpoC	DNA-directed RNA polymerase subunit beta
CW_00060	CDS	56696	60589	-	rpoB	DNA-directed RNA polymerase subunit beta
CW_00061	CDS	60567	60929	-	rplL	50S ribosomal protein L7/L12
CW_00062	CDS	60956	61423	-	rplJ	50S ribosomal protein L10
CW_00063	CDS	61441	62094	-	rplA	50S ribosomal protein L1
CW_00064	CDS	62084	62509	-	rplK	50S ribosomal protein L11
CW_00065	tRNA	62796	62879	-		tRNA-Tyr
CW_00066	tRNA	62888	62962	-		tRNA-Thr
CW_00067	CDS	62987	63190	-	rpmE	50S ribosomal protein L31
CW_00068	CDS	63228	63746	+	hslV	ATP-dependent protease subunit hslV
CW_00069	CDS	63882	65102	+	hslU	ATP-dependent protease ATPase subunit hslU
CW_00070	CDS	65113	65334	+	rpsU	30S ribosomal protein S21
CW_00071	CDS	66631	67326	+	rpoD	RNA polymerase sigma factor rpoD
CW_00072	CDS	67361	68212	-	cyoE	protoheme IX farnesyltransferase
CW_00073	CDS	68519	69082	-	cyoC	cytochrome o ubiquinol oxidase, subunit III
CW_00074	CDS	69086	70906	-	cyoB	cytochrome o ubiquinol oxidase, subunit I
CW_00075	CDS	71241	71909	-	cyoA	cytochrome o ubiquinol oxidase subunit II
CW_00076	CDS	71921	72409	-	ruvC	crossover junction endodeoxyribonuclease RuvC
CW_00077	CDS	72429	73235	+	proC	pyrroline-5-carboxylate reductase
CW_00078	tRNA	73236	73309	-		tRNA-Arg
CW_00079	CDS	73431	74090	-	truA	tRNA pseudouridine(38-40) synthase TruA
CW_00080	CDS	74120	75847	+	mnmG	tRNA uridine-5-carboxymethylaminomethyl(34) synthesis enzyme MnmG

CW_00081	CDS	75873	76676	+	atpB	F0F1 ATP synthase subunit A
CW_00082	CDS	76707	76940	+	atpE	F0F1 ATP synthase subunit C
CW_00083	CDS	76941	77411	+	atpF	F0F1 ATP synthase subunit beta
CW_00084	CDS	77971	79509	+	atpA	F0F1 ATP synthase subunit alpha
CW_00085	CDS	79513	80361	+	atpG	F0F1 ATP synthase subunit gamma
CW_00086	CDS	80367	81740	+	atpD	F0F1 ATP synthase subunit beta
CW_00087	CDS	81742	81987	+	atpC	F0F1 ATP synthase subunit epsilon
CW_00088	tRNA	81989	82061	+		tRNA-Ile
CW_00089	CDS	82366	82581	+	rpsR	30S ribosomal protein S18
CW_00090	tRNA	82656	82742	+		tRNA-Ser
CW_00091	tRNA	82763	82835	+		tRNA-Arg
CW_00092	CDS	82864	83106	+	rpsP	30S ribosomal protein S16
CW_00093	CDS	83113	83424	+	rplS	50S ribosomal protein L19
CW_00094	CDS	83429	84346	+	htpX	Peptidase family M48
CW_00095	CDS	84385	84915	+	infC	translation initiation factor IF-3
CW_00096	CDS	84908	85093	+	rpmI	50S ribosomal protein L35
CW_00097	CDS	85095	85457	+	rplT	50S ribosomal protein L20
CW_00098	tRNA	85490	85573	+		tRNA-Leu
CW_00099	tRNA	85599	85670	+		tRNA-Gln
CW_00100	tRNA	85705	85779	+		tRNA-Met
CW_00101	CDS	85830	86900	+	miaB	tRNA-N(6)-(isopentenyl)adenosine-37 thiotransferase MiaB
CW_00102	CDS	87136	88710	-	groEL	chaperonin GroEL
CW_00103	CDS	88732	89007	-	groES	co-chaperonin GroES
CW_00104	CDS	89032	90285	-	dnaB	replicative DNA helicase DnaB
CW_00105	CDS	90647	91072	-		ribonuclease III family protein
CW_00106	CDS	91074	92840	-	lepA	Translation elongation factor LepA
CW_00107	CDS	92842	94053	-	fabB	beta-ketoacyl-ACP synthase I
CW_00108	tRNA	94821	94907	+		tRNA-Ser
CW_00109	CDS	94909	95955	+	prfA	peptide chain release factor 1
CW_00110	CDS	95961	96884	-	chaA	sodium-potassium/proton antiporter ChaA
CW_00111	CDS	97520	98938	-	lysS	lysine--tRNA ligase
CW_00112	CDS	98971	100752	+	ftsH	ATP-dependent zinc metalloprotease FtsH
CW_00113	tRNA	101137	101210	+		tRNA-Met
CW_00114	CDS	101327	102808	+	infB	translation initiation factor IF-2
CW_00115	CDS	102810	103064	+	rpsO	30S ribosomal protein S15
CW_00116	CDS	103522	103884	-	smpB	SsrA-binding protein SmpB
CW_00117	CDS	103932	104528	-	ribE	riboflavin synthase subunit alpha
CW_00118	CDS	104613	105854	+	matE	MATE family efflux transporter
CW_00119	CDS	105945	106319	+		hypothetical protein
CW_00120	CDS	106322	107008	+	dnaQ	DNA polymerase III subunit epsilon
CW_00121	CDS	107093	107701	+	rpsB	30S ribosomal protein S2
CW_00122	CDS	107707	108519	+	tsf	translation elongation factor Ts
CW_00123	CDS	108525	109094	+		hypothetical protein
CW_00124	CDS	109096	111858	+	dnaE	DNA polymerase III subunit alpha
CW_00125	CDS	112063	112497	+		hypothetical protein
CW_00126	CDS	112489	112701	-		signal peptidase II
CW_00127	CDS	113465	115699	-	ileS	isoleucine--tRNA ligase
CW_00128	CDS	115739	116005	+	rpsT	30S ribosomal protein S20
CW_00129	CDS	116083	117210	-	dnaJ	molecular chaperone DnaJ

CW_00130	CDS	117354	119267	-	dnaK	molecular chaperone DnaK
CW_00131	tRNA	119314	119385	+		tRNA-Glu
CW_00132	CDS	119453	120535	+	brnQ	branched-chain amino acid transport system II carrier protein
CW_00133	tRNA	121106	121193	-		tRNA-Ser
CW_00134	CDS	121519	121773	+	rpmA	50S ribosomal protein L27
CW_00135	CDS	121775	122767	+	obgE	GTPase ObgE
CW_00136	CDS	122972	124534	+	cysJ	NADPH-dependent assimilatory sulfite reductase flavoprotein subunit
CW_00137	CDS	124535	126139	+	cysI	NADPH-dependent assimilatory sulfite reductase hemoprotein subunit
CW_00138	CDS	126244	126891	+	cysD	phosphoadenosine phosphosulfate reductase
CW_00139	CDS	126974	128311	+	cysG	siroheme synthase CysG
CW_00140	CDS	128336	129328	-	trpS	tryptophan--tRNA ligase
CW_00141	CDS	129333	130955	-	glyS	glycine--tRNA ligase subunit beta
CW_00142	CDS	131263	131946	-	glyQ	glycine--tRNA ligase subunit alpha
CW_00143	CDS	132709	134949	-	leuS	leucine--tRNA ligase
CW_00144	CDS	134978	135412	+	def	peptide deformylase
CW_00145	CDS	135412	136338	+		methionyl-tRNA formyltransferase
CW_00146	CDS	136341	136697	-	rplQ	50S ribosomal protein L17
CW_00147	CDS	136728	137735	-	rpoA	DNA-directed RNA polymerase subunit alpha
CW_00148	CDS	137737	138330	-	rpsD	30S ribosomal protein S4
CW_00149	CDS	138361	138699	-	rpsK	30S ribosomal protein S11
CW_00150	CDS	139604	140008	-	rpsM	30S ribosomal protein S13
CW_00151	CDS	140131	141441	-	secY	preprotein translocase subunit SecY
CW_00152	CDS	141449	141871	-	rplO	50S ribosomal protein L15
CW_00153	CDS	141861	142355	-	rpsE	30S ribosomal protein S5
CW_00154	CDS	142378	142725	-	rplR	50S ribosomal protein L18
CW_00155	CDS	142727	143119	-	rplF	50S ribosomal protein L6
CW_00156	CDS	143275	143664	-	rpsH	30S ribosomal protein S8
CW_00157	CDS	143669	143971	-	rpsN	30S ribosomal protein S14
CW_00158	CDS	143975	144520	-	rplE	50S ribosomal protein L5
CW_00159	CDS	144524	144832	-	rplX	50S ribosomal protein L24
CW_00160	CDS	144837	145205	-	rplN	50S ribosomal protein L14
CW_00161	CDS	145244	145453	-	rpsQ	30S ribosomal protein S17
CW_00162	CDS	145627	146043	-	rplP	50S ribosomal protein L16
CW_00163	CDS	146045	146656	-	rpsC	30S ribosomal protein S3
CW_00164	CDS	146722	147048	-	rplV	50S ribosomal protein L22
CW_00165	CDS	147032	147397	-	rpsS	30S ribosomal protein S19
CW_00166	CDS	147477	148220	-	rplB	50S ribosomal protein L2
CW_00167	CDS	148234	148521	-	rplW	50S ribosomal protein L23
CW_00168	CDS	148524	149132	-	rplD	50S ribosomal protein L4
CW_00169	CDS	149140	149766	-	rplC	hypothetical protein
CW_00170	CDS	149766	150077	-	rpsJ	30S ribosomal protein S10
CW_00171	CDS	150079	151257	-		elongation factor EF-Tu
CW_00172	CDS	151259	153361	-	fusA	elongation factor G
CW_00173	CDS	153420	153887	-	rpsG	30S ribosomal protein S7
CW_00174	CDS	153880	154266	-	rpsL	30S ribosomal protein S12
CW_00175	CDS	154441	155895	-	metG	methionine--tRNA ligase

CW_00176	CDS	155888	156925	-	rlmN	23S rRNA (adenine(2503)-C(2))-methyltransferase RlmN
CW_00177	CDS	156961	158229	+	der	ribosome biogenesis GTPase Der
CW_00178	tRNA	158236	158308	-		tRNA-Phe
CW_00179	tRNA	158324	158396	-		tRNA-Pro
CW_00180	tRNA	158404	158474	-		tRNA-His
CW_00181	CDS	158494	158832	-	trxA	thioredoxin TrxA
CW_00182	CDS	158846	159625	-	rsmA	16S rRNA (adenine(1518)-N(6)/adenine(1519)-N(6))-dimethyltransferase RsmA
CW_00183	CDS	159609	160259	-	lgt	prolipoprotein diacylglyceryl transferase
CW_00184	CDS	162251	163867	+	glnS	glutamine--tRNA ligase
CW_00185	CDS	163873	164133	-	infA	translation initiation factor IF-1
CW_00186	CDS	164138	164533	-	ruvX	Holliday junction resolvase RuvX
CW_00187	CDS	164646	165638	+	mnmA	tRNA 2-thiouridine synthase MnmA

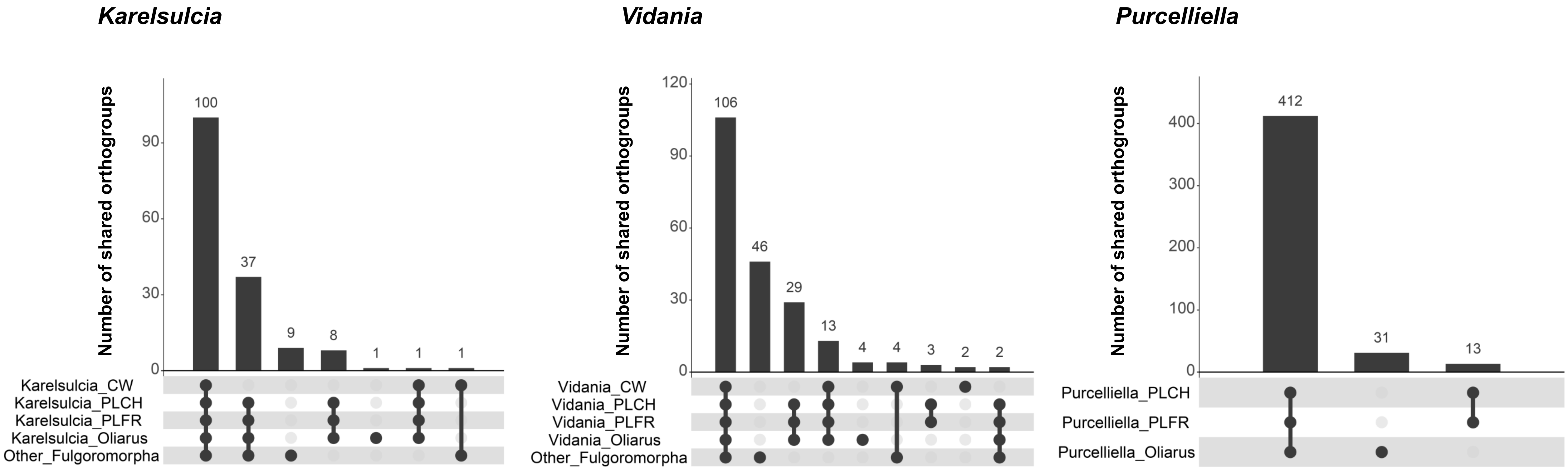

Supplementary Figure S1. Intersection plots showing the amount of shared orthogroups between the endosymbionts of *Cixius wagneri* and *Pentastiridius leporinus* with the corresponding endosymbionts from *Oliarus* spp. (i.e. strains OLIH, OFIL1, OFIL2, OPOL1, OPOL2) and from other Fulgoromorpha (i.e. the strains CALKRU, DICMUL, RANSCY, PYRCLA, PYRLAN, PYRVIR).

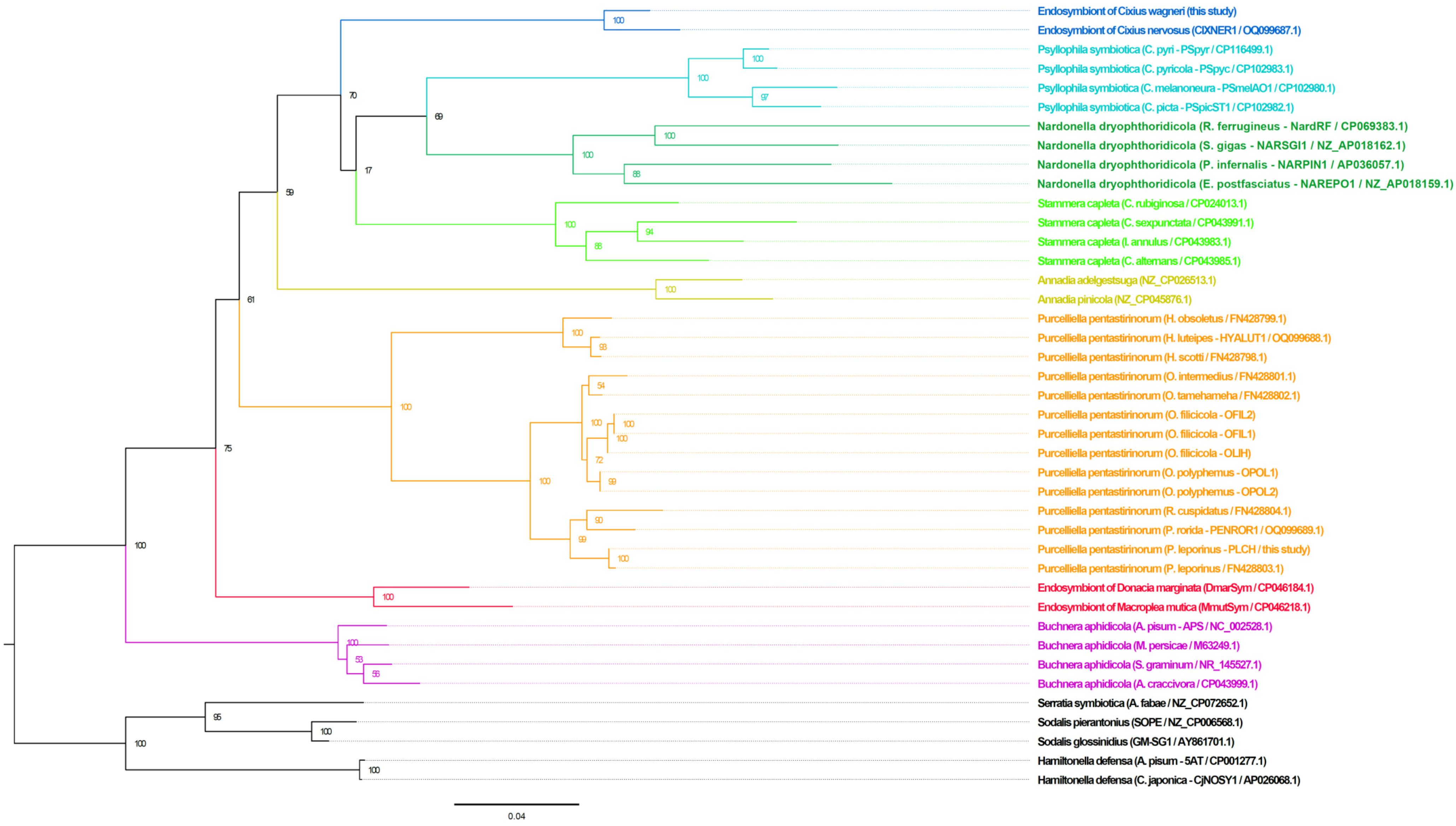

Supplementary Figure S2. Maximum-likelihood phylogenetic tree based on the 16S rRNA genes of 41 nutritional endosymbionts from the *Gammaproteobacteria*, with *Sodalis* spp., *Serratia symbiotica* and *Hamiltonella defensa* as outgroup. Colours indicate different genera. The endosymbionts of *C. wagneri* and *C. nervosus* cluster together and represent a new species-level clade. Branch support is based on 1000 bootstrap iterations.

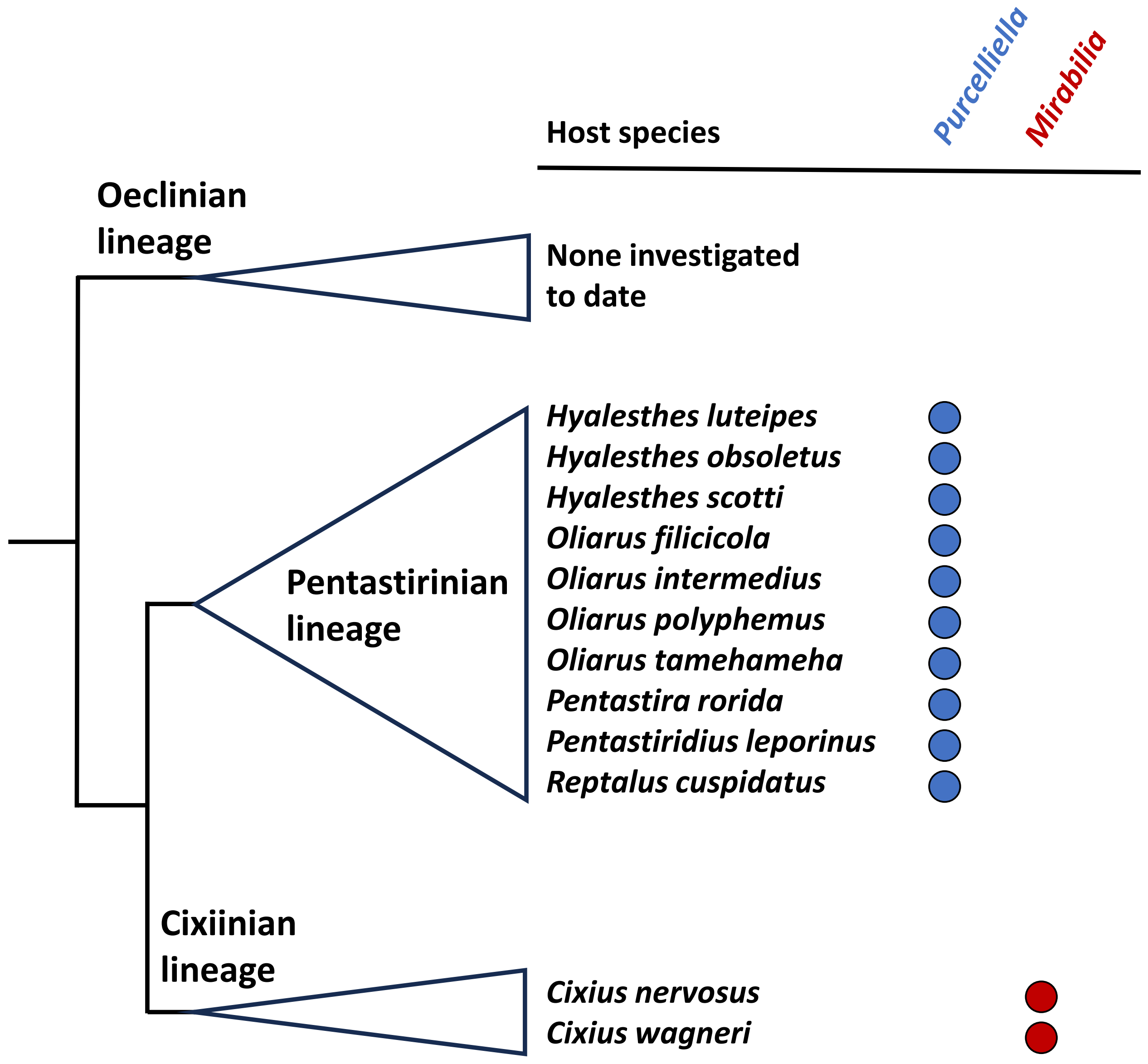

Supplementary Figure S3. Schematic representation of *Gammaproteobacteria* endosymbiont distribution across all Cixiidae species investigated to date. Subfamily lineages are based on (79). For each lineage, the investigated host species and their *Gammaproteobacteria* endosymbionts are shown. Endosymbiont data is based on (10, 29, 30, 41) and this study.
